# Assessing Team Effectiveness by How Players Structure Their Search in a First‐Person Multiplayer Video Game

**DOI:** 10.1111/cogs.13204

**Published:** 2022-10-17

**Authors:** Patrick Nalepka, Matthew Prants, Hamish Stening, James Simpson, Rachel W. Kallen, Mark Dras, Erik D. Reichle, Simon G. Hosking, Christopher Best, Michael J. Richardson

**Affiliations:** ^1^ School of Psychological Sciences Macquarie University; ^2^ Centre for Elite Performance, Expertise and Training Macquarie University; ^3^ School of Computing Macquarie University; ^4^ Human and Decision Sciences Division Defence Science and Technology Group

**Keywords:** Collective human behavior, Search behaviors, Division of labor, Situation awareness, Detrended fluctuation analysis, head‐up displays

## Abstract

People working as a team can achieve more than when working alone due to a team's ability to parallelize the completion of tasks. In collaborative search tasks, this necessitates the formation of effective division of labor strategies to minimize redundancies in search. For such strategies to be developed, team members need to perceive the task's relevant components and how they evolve over time, as well as an understanding of what others will do so that they can structure their own behavior to contribute to the team's goal. This study explored whether the capacity for team members to coordinate effectively can be related to how participants structure their search behaviors in an online multiplayer collaborative search task. Our results demonstrated that the structure of search behavior, quantified using detrended fluctuation analysis, was sensitive to contextual factors that limit a participant's ability to gather information. Further, increases in the persistence of movement fluctuations during search behavior were found as teams developed more effective coordinative strategies and were associated with better task performance.

## 1 Introduction

Teams can accomplish more than individuals working alone due to a team's ability to divide labor among members to parallelize the completion of tasks (Brennan, Chen, Dickinson, Neider, & Zelinsky, 2008; Goldsby, Dornhaus, Kerr, & Ofria, 2012; Goldstone & Gureckis, 2009). The benefit of working within a larger group structure is most apparent in collaborative search tasks, such as the physical search for food or other items (Brennan et al., 2008; Goldstone & Gureckis, 2009; Wu et al., 2021), or in conceptual search such as finding the maximum payout in a multi‐armed bandit problem (Toyokawa, Whalen, & Laland, 2019; Wu, Schulz, Speekenbrink, Nelson, & Meder, 2018). In these settings, searching collaboratively not only allows groups to search in parallel but the observation of others’ actions can also inform how individuals should search (Wu et al., 2021). However, despite the many benefits of working collaboratively, success in teams is not guaranteed (Roberts & Goldstone, 2011; Toyokawa et al., 2019) due to the challenges associated with coordinating actions that align with the team's objective. To overcome these challenges, team members must exchange information through dialogue (Coco, Dale, & Keller, 2018; Fusaroli et al., 2012) or perceptual coupling (Hoehl, Fairhurst, & Schirmer, 2021) to both reduce redundant work and allow individuals to effectively plan their own actions.

In the teamwork literature, the capacity for teams to coordinate and work effectively requires each team member to develop the situation awareness (SA) required for their job. The term “situation awareness”, historically, has been defined as “the perception of elements in the environment within a volume of time and space, the comprehension of their meaning, and the projection of their status in the near future” (Endsley, 1988, p. 97; see also Gorman, Cooke, & Winner, 2006). Within a team context, team SA (or TSA) refers to each team member possessing “the situation awareness required for his/her job” (Endsley, 1995; see also Demir, McNeese, & Cooke, 2017; McNeese, Demir, Cooke, & She, 2021). Because teams are interactive in nature, this also includes individuals understanding when and to whom information should be shared to facilitate team coordination (Cooke & Gorman, 2009).

Inspired by the collective behavior observed in collaborative search and herding in animals, humans, and other multiagent systems (Nalepka et al., 2017; Nalepka, Silva et al., 2021), this paper explores the development of skilled performance in three‐person teams engaged in the physical search and retrieval of autonomous objects in a large virtual environment. This paper explores whether the development of effective team division‐of‐labor strategies can be assessed by how team members structure their individual search behavior to establish SA. We manipulate the ability to develop SA by manipulate the ease in obtaining task‐relevant information—either by altering the visibility of the environment, or augmenting information presented via a head‐up display (HUD). Elaborated in more detail below, search structure will be assessed by analyzing the fluctuations of individual perceptual‐motor behavior using detrended fluctuation analysis (DFA) (Hardstone et al., 2012; Peng et al., 1994). Variation in DFA has been theorized to reflect the degree to which individuals can control and anticipate the dynamics of their environment—a key component for SA to be possible. In conjunction with other techniques to assess the ongoing coordination of teams (e.g., Gorman et al., 2020), DFA can provide another tool to quantify the development of taskwork and teamwork skills in individual team members.

## 2 Background

### 2.1 Context sensitivity of search behaviors

Effective search strategies in animals, including humans, reflect the interaction between the complexity of the search environment (i.e., its predictability) (Hills, Kalff, & Wiener, 2013; Humphries et al., 2010) and the capabilities of the searcher (Nauta, Khaluf, & Simoens, 2020). For example, in search tasks where the item to locate is sparsely populated, behaviors that exhibit fractal, Lévy‐like paths are found to be optimal (Viswanathan et al., 1999). These paths are characterized by local, exhaustive search interspersed with far relocations and have been observed in several animal species (Viswanathan et al., 1999), including humans (Raichlen et al., 2014). This search process is regarded as memory‐less and entails choosing a random heading direction at each displacement. When the search items are abundant in the environment, or if there is knowledge as to where the items are located, then exhaustive local search is more efficient (Hills et al., 2013; Humphries et al., 2010; Nauta et al., 2020). In addition to environmental and physical constraints, task constraints also impact the search strategies that humans use to locate items, such as the necessity to return to a home location (Garg & Kello, 2021). Outside of physical search, similar patterns of “optimal” behavior are observed during search in conceptual spaces like memory recall (Hills et al., 2015; Patten, Greer, Likens, Amazeen, & Amazeen, 2020; Szary, Dale, Kello, & Rhodes, 2015).

### 2.2 Exploratory search behaviors and team performance

The adaptive nature of search behaviors has also been observed in team‐based sports contexts, where scanning behaviors by players serve a functional role in maintaining SA. Research investigating soccer athletes has demonstrated that the extent to which players perform exploratory movements (assessed by variability in head orientation) is related to player expertise (McGuckian, Cole, Chalkley, Jordet, & Pepping, 2020; Phatak & Gruber, 2019), as well as certain task contextual factors which elicit a greater need for SA (McGuckian et al., 2020). For example, the degree to which players scanned their surrounding environment was associated with better passing accuracy and lower turnover rate. Additionally, greater head movements were found to occur when players were in midfield positions, when in possession of the soccer ball, or when near either goal.

The function of athletes performing exploratory search behaviors is to establish greater SA to enhance task performance. Indeed, limiting an individual's ability to perform these exploratory behaviors, or depriving access to task‐relevant information, has been demonstrated to reduce task performance (e.g., Alfano & Michel, 1990; Crebolder & Sloan, 2004; Fransen et al., 2017; Ragan et al., 2015; Toet, Jansen, & Delleman, 2007; Wood & Troutbeck, 1994; Wood, Chaparro, Carberry, & Chu, 2010). For example, Fransen et al. (2017) found that dribbling performance by soccer players is severely reduced when visual feedback is withheld. Likewise, obstructed vision has been found to decrease SA, and thus performance, in a variety of other sporting contexts. This has resulted in the emergence of training paradigms designed to reduce players reliance on visual information (Dunton, O'Neill, & Coughlan, 2019; Hülsdünker et al., 2019; Oudejans, 2012).

### 2.3 Measuring situation awareness via the structure of behavior

Not only is the magnitude of search important (e.g., standard deviation of head angular movements [McGuckian et al., 2020; Phatak & Gruber, 2019]), but how this search is structured to facilitate adaptive behavior is equally important (e.g., as demonstrated by Garg & Kello, 2021). That is, how the variability of behavior is structured across time can provide valuable information about the organization of the underlying cognitive system (Gilden, 2001). Specifically, variability that is time‐independent (i.e., white noise) arises from component‐driven dynamics and is the result of a linear accumulation of error during cognitive processing (van Orden, Holden, & Turvey, 2003). In contrast, variability which is time‐dependent emerges from the dynamic interaction of system components (e.g., pink noise, also referred to as 1/*f* noise) and is characteristic of interaction‐dominant or self‐organizing systems. These systems exhibit self‐similar patterns (i.e., fractal) of variability across timescales of observation and have been observed in a number of human physiological processes and cognitive behaviors (e.g., for a review, see Kello et al., 2010). The seeming ubiquity of fractal‐like variability in human behavior suggests that cognitive systems operate near states of self‐organized criticality (Bak, 1996; Kello et al., 2010). Systems near critical states are most responsive to perturbations (i.e., they exhibit metastability; Tognoli & Kelso, 2014), and thus are maximally adaptive (Kello et al., 2010).

**Fig. 1:**
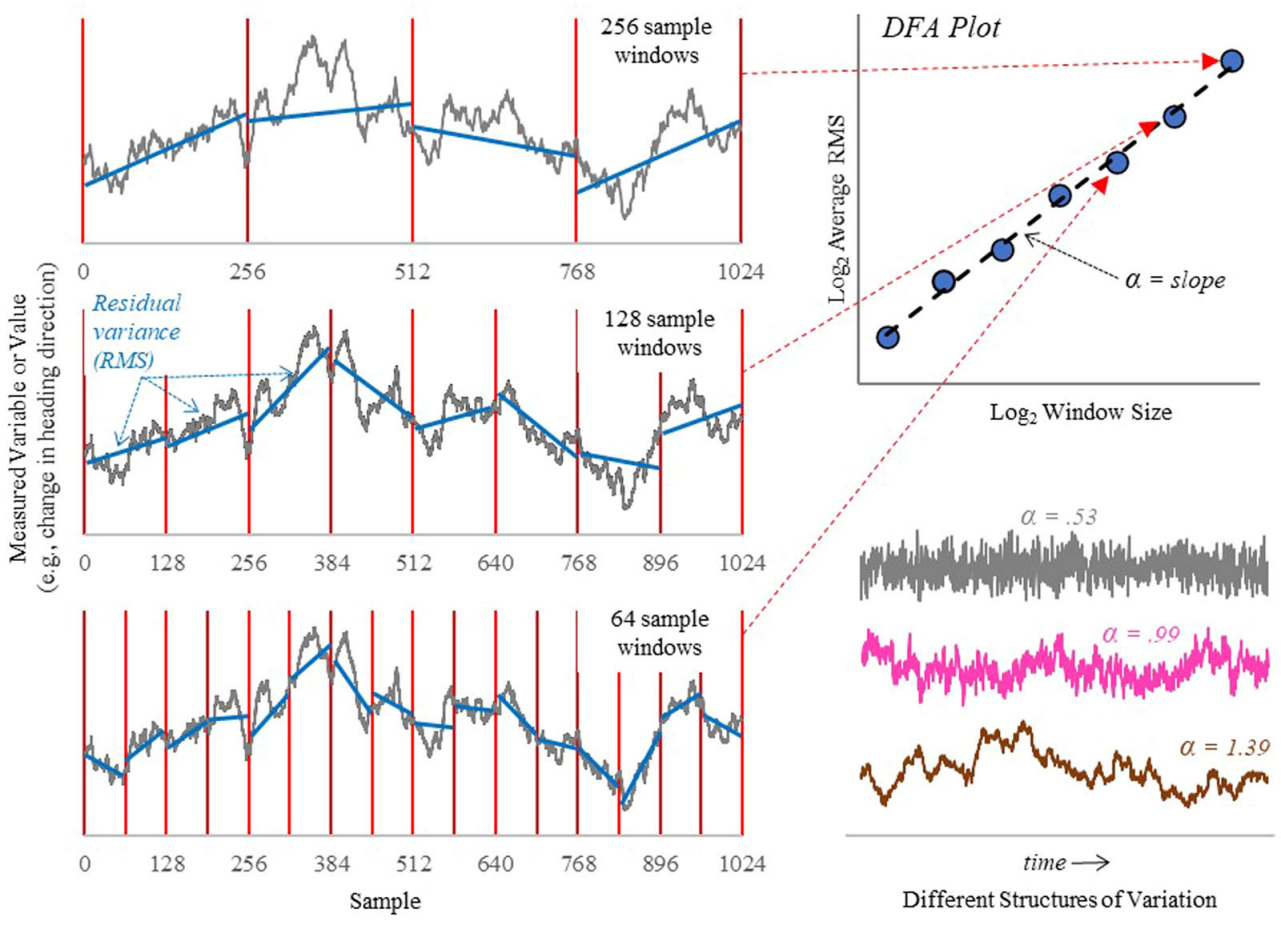
Illustration of DFA. DFA involves the measurement of detrended, residual variance of fluctuation time series at different time windows. The figure demonstrates the procedure over window sizes spanning 64, 128, and 256 samples. Within each window, the time series is detrended, and the residual variance is calculated and averaged across all windows and plotted on a log–log plot (top right). The slope of the best‐fit line provides an estimate of the variability structure. Examples of white, pink, and brown noise are shown with respective DFA_α_ values (bottom right). Adapted from Rigoli, Lorenz, et al. (2020).

A common method to quantify the structure of variability is DFA (Peng et al., 1994). An illustration for how DFA is conducted is shown in Fig. 1. Given a time series of behavior fluctuations, DFA involves linearly detrending the signal at various window sizes, averaging the residual variance across windows of a similar length, and then plotting the resultant value on a log–log plot for each window size. The slope of the resulting best‐fit line provides an estimate for how variability is structured across time (where the slope is represented as DFA_α_). Variability that is time‐independent, and therefore unstructured, results in slope values ≈ 0.5. Slope values < 0.50 are indicative of an antipersistent signal that is reactive to previous inputs (e.g., such as tapping to a metronome; Chen, Ding, & Scott Kelso, 2001). Slope values > 0.50 and approaching 1.00 indicate persistence in the variability of the time series (i.e., positive [negative] deviations are more likely to follow positive [negative] deviations). A slope of 1.00 indicates fractal structure (i.e., pink noise). Slopes < 1.00 indicate the time series is a fractional, Gaussian process which is stationary. DFA_α_ > 1.00 indicate a non‐stationary, fractional Brownian process, where the variability time series exhibits long system “memory,” whereby the variation at any timepoint is heavily influenced by the system's history.

The value of DFA_α_ is sensitive to experimental manipulation and is theorized to reflect an interaction between individual agency and control, with constraints provided by the environment and task context (Kello, Beltz, Holden, & van Orden, 2007; van Orden, Kloos, & Wallot, 2011). Using the example of component‐dominant versus interaction‐dominant dynamics from above, tasks which exhibit strong external constraints (e.g., unpredictable stimulus onset; Kello et al., 2007) produce behavior fluctuations which are random/time‐independent. When the constraints from the environment are lessened, such as through greater control during skill development (e.g., Stallworthy et al., 2020), behavior fluctuations become more interdependent which reflects greater coupling between the agent with their environment (i.e., interaction‐dominant dynamics resulting in fractal behavior) (Dotov, Nie, & Chemero, 2010). DFA_α_ can be understood as reflecting a scale that balances external, environment, and task constraints, with internal, individual constraints. Variation in DFA_α_ due to the interplay of task, environment, and internal constraints also extends to social contexts, where a phenomenon known as “complexity matching” can occur (Abney, Paxton, Dale, & Kello, 2014; Almurad, Roume, & Delignières, 2017; Rigoli, Lorenz, et al., 2020), where the DFA_α_ of coactors can be found to correlate with one another, possibly to enable maximal information transmission between individuals (West, Geneston, & Grigolini, 2008).

The interaction between external versus internal sources of constraints and behavioral variability are highlighted in several studies (Dingwell & Cusumano, 2010; Irrmischer, van der Wal, Mansvelder, & Linkenkaer‐Hansen, 2018; Likens, Fine, Amazeen, & Amazeen, 2015; Washburn, Coey, Romero, Malone, & Richardson, 2015). Critically, the variation of constraints on the variation in behavior can be studied along task‐relevant and task‐irrelevant components (Dingwell & Cusumano, 2010; Kello et al., 2007; Washburn et al., 2015), which allows for the differentiation in the role of dimension‐specific constraints on variability within the same session. For example, in a study where participants were required to walk on a treadmill at different speeds, the variation in walking speed exhibited antipersistent behavior, as that was the component of movement that was task‐controlled, while components such as stride length or stride time were not constrained by such demands and were characterized by more fractal (i.e., DFA_α_ > 0.5) behavioral variability (Dingwell & Cusumano, 2010). In a speeded response task where participants had to indicate if an image appeared for a longer than expected duration, the persistence in participant responses increased when participants were mind wandering—indicative of a balance tilted towards internal, individual constraints (Irrmischer et al., 2018). And finally, in a rhythmic‐movement task where participants controlled either the period or amplitude of their movement, the introduction of a visual stimulus to facilitate pacing resulted in decreases in DFA_α_ scaling (Washburn et al., 2015). Similarly, Likens et al. (2015) demonstrated that in a simple driving task, participants exhibited more persistent, fractal behavior when participants drove on a circular track, but exhibited less persistent, corrective behavior when driving on the “easier” straight path. Consistent with the views of van Orden et al. (2011), the findings of Likens et al. (2015) indicate that a lack of task engagement or necessity for voluntary control in continuous control problems can “shift” the balance away from internal sources of control (due to disengagement) towards environmental constraining forces.

Regarding SA, the latter research suggests that changes in DFA_α_ could also indicate the extent to which individuals are coupled to and situationally aware of their surrounding environment. Indeed, the sensitivity of DFA_α_ to changes with and across task contexts and the concept of SA are already conceptually similar. That is, changes in DFA_α_ (the fractality of behavior) indicate changes in an individual's ability to plan and execute actions effectively and in a prospective, not reactive, manner. Analogously, individuals are said to possess SA if they can perceive and anticipate how a task will evolve over time—to effectively plan their own behavior.

### 2.4 The use of assistive technologies to facilitate team coordination

Most real‐world search task contexts involve the detection of task‐relevant information from a first‐person point of observation. Accordingly, individuals who more frequently search their environment for task‐relevant information develop greater SA of the task, which typically results in better performance and more task‐specific expertise (McGuckian et al., 2020; Phatak & Gruber, 2019). However, modern motion tracking, wearable and smartphone GPS and location‐tracking technologies, augmented reality, and HUD technologies now mean that in many team contexts there is a growing potential for team members to have access to more “global” task state information, enabling team members to obtain greater SA more easily. Augmenting access to task‐relevant information via the use of assistive technologies has been found to improve task performance (Smith, Streeter, Burnett, & Gabbard, 2015; Sojourner & Antin, 1990; Ververs & Wickens, 2000). For example, Charissis et al. (2008) found that participants performed better in a simulated driving task when they were provided with a HUD that gave them additional information about the task environment beyond their own perspective. Similarly, it was found that providing pilots with HUD glasses that highlighted air traffic pathways within their flying area led to an increased ability to detect and react to possible obstacles (Rafi, Chandrasekaran, Kusmez, Steck, & He, 2018). The use of “shared displays” is also common in the teams literature to facilitate TSA (Bolstad & Endsley, 1999).

The use of assistive technologies in facilitating SA is perhaps most readily experienced when playing any modern multiplayer video game, where players often have access to a HUD indicating the relative or absolute location of other players, opponents, or objectives. In real‐time strategy (RTS) games, players prefer gaming on large displays that enable more of the game environment to be displayed to reduce exploratory behaviors, where decreases in exploration were associated with greater performance (Sabri, Ball, Fabian, Bhatia, & North, 2007). In first‐person multiplayer shooters (FPS), HUDs facilitate game play by graphically presenting and summarizing key task‐relevant information that falls outside a player's first‐person point of view (Zammitto, 2008). The more effective the HUD is in achieving this, the more likely the players are to employ it (Jørgensen, 2012). Indeed, the use of the HUD often becomes key to how players engage and play video games. For instance, Caroux and Isbister (2016) have demonstrated how in both RTS and FPS games players spend a significant amount of time viewing a HUD and that access to a permanent HUD improved players’ understanding of the task environment. In particular, RTS players spent on average just under one quarter of game time viewing the HUD, and experts spent more time utilizing the HUD than novices. What this indicates is that video game players rely heavily on the HUD to facilitate behavior, and this reliance increases with experience.

### 2.5 Current study

This study explored team collaboration in a three‐person search and retrieval task that involved locating a set of autonomous agents and corralling them to a central containment region. The task was inspired by previous research exploring corralling behaviors in two‐person task contexts (Nalepka et al., 2017, 2019, Nalepka, Silva, et al., 2021; Rigoli, Nalepka, et al., 2020). The task was developed as an online, multiplayer video game where participants could navigate a large desert environment using keyboard and mouse controls like what is used in commercial, FPS games. Teams were recruited to play this game across four sessions. The game was presented as separate 5‐min trials, and teams had to coordinate to locate and contain either 9 or 18 autonomous agents within the time limit. Teams were incentivized to complete each trial as quickly as possible with the fastest performing team receiving a monetary prize at the last session.

We introduced aspects of the task environment to manipulate the ease for team members to develop SA. First, the level of visibility while completing the task was manipulated to either allow for good or poor visibility (>150 m and approximately 10 m, respectively) while navigating the desert terrain. Second, the availability of global, task‐relevant information to facilitate participant decision‐making was manipulated via the use of a HUD provided to participants. The HUD was presented either as a compass, where participants could obtain their direction of heading as well as position in reference to the containment region, or it contained information regarding all task‐relevant components—the position and heading direction of all team members, the location of all target agents (TAs), and the state of these agents (i.e., whether they were idle, fleeing, or within the containment region).

With experience and when teams could easily access task‐relevant information, teams were expected to divide task labor more effectively by reducing the redundancy of search in the task environment. Further, the structure of participant search behaviors was expected to be modulated by the availability or ease in obtaining task‐relevant information to structure their movements across the desert environment. Specifically, when participants could easily obtain information from their surrounding environments, participants were expected to exhibit greater structure in variability in their search movement (i.e., high values of DFA_α_)—indicative of prospective planning and control (i.e., clear visibility, with access to global HUD information). When participants lacked this information, or when participants were required to perform search behaviors to obtain task‐relevant information, participants were expected to show decreased structure in their search and movement behaviors (i.e., lower values of DFA_α_) due to participants needing to react to information that is uncovered during search (e.g., when visibility was poor or team members were only given a compass).

## 3 Method

### 3.1 Recruitment criteria

Recruitment materials were distributed via e‐mail to senior undergraduate psychology, cognitive science, and computer science students at Macquarie University and via social media posts to groups targeting undergraduate students. Interested individuals were asked to complete an interest survey where demographic information (e.g., age, gender), availability (e.g., days/times during the week), computer hardware and software specifications, and Internet speed information were collected. From those who completed the interest survey, potential participants were contacted who met the following criteria: their computer hardware and software met minimum specifications (Intel i5 [7th generation] or equivalent CPU, Intel HD Graphics 500 or equipment GPU, 8 GB RAM, 1280 × 720 or greater screen resolution, Windows 10 or MacOS 10.14 Mojave operating systems); and they had an internet connection with a download speed greater than 5 Mb⋅s^–1^, with a ping of <100 ms (http://www.speedtest.net). A total of 102 individuals met these minimum requirements.

### 3.2 Participants

Thirty individuals (16 female, 14 male) were pseudorandomly selected to participate in the study. Individuals were placed into 10, three‐person teams with similar day/time availability. Teams consisted of at least one female (five teams with one female and two males; four teams with two females and one male; one team with three females). Participants ranged in age from 18 to 45 years (*M* = 23.19, *SD* = 4.71), and all participants were either native (*N* = 24) or fluent (*N* = 6) English language speakers. None of the participants knew their teammates prior to participating in the experiment, and all were naïve to the purpose of the study. All participants completed the experiment remotely online using their own personal/home computer running Windows or MacOS operating systems. Participants completed the experiment across four sessions, with each session being approximately 1.5 h in duration. Sessions were conducted once per week (*M* = every 6.87 days, *SD* = 3.17). Participants received $30 AUD for each session, and a bonus of $40 AUD for completing all four sessions with the potential to receive an additional $50 AUD if their team had the best performance in Session 4 (assessed as the team who completed the most trials successfully and most rapidly in the case of a tie). Payment was provided in the form of an e‐Gift Card. The recruitment procedures and study methodology were approved by the Macquarie University Human Research Ethics Committee.

### 3.3 Materials and design

#### 3.3.1 Desert Herding game

Across all sessions, teams played a networked multiplayer game referred to as Desert Herding. The game was designed using the Unity3D Game Engine (version 2018.4 LTS; Unity Technologies, San Francisco, CA). The game server was hosted on Amazon Web Services EC2 with Windows 10. Participants downloaded a standalone version of the game to their personal/home computer and connected to the server as clients. Game states, including participant movements, were transmitted across the network using Mirror (vis2k, https://github.com/vis2k/Mirror/) networking architecture. The game could only be played when the experimenter instantiated a game session on the server, and thus participants could only play the game and complete the experiment during their allocated sessions. The server recorded all game state data at a sample rate of 90 Hz. In addition to interacting with each other in‐game, participants could verbally communicate via the use of Internet teleconferencing software (Zoom Video Communications Inc., San Jose, CA; set to audio‐only communication).

**Fig. 2:**
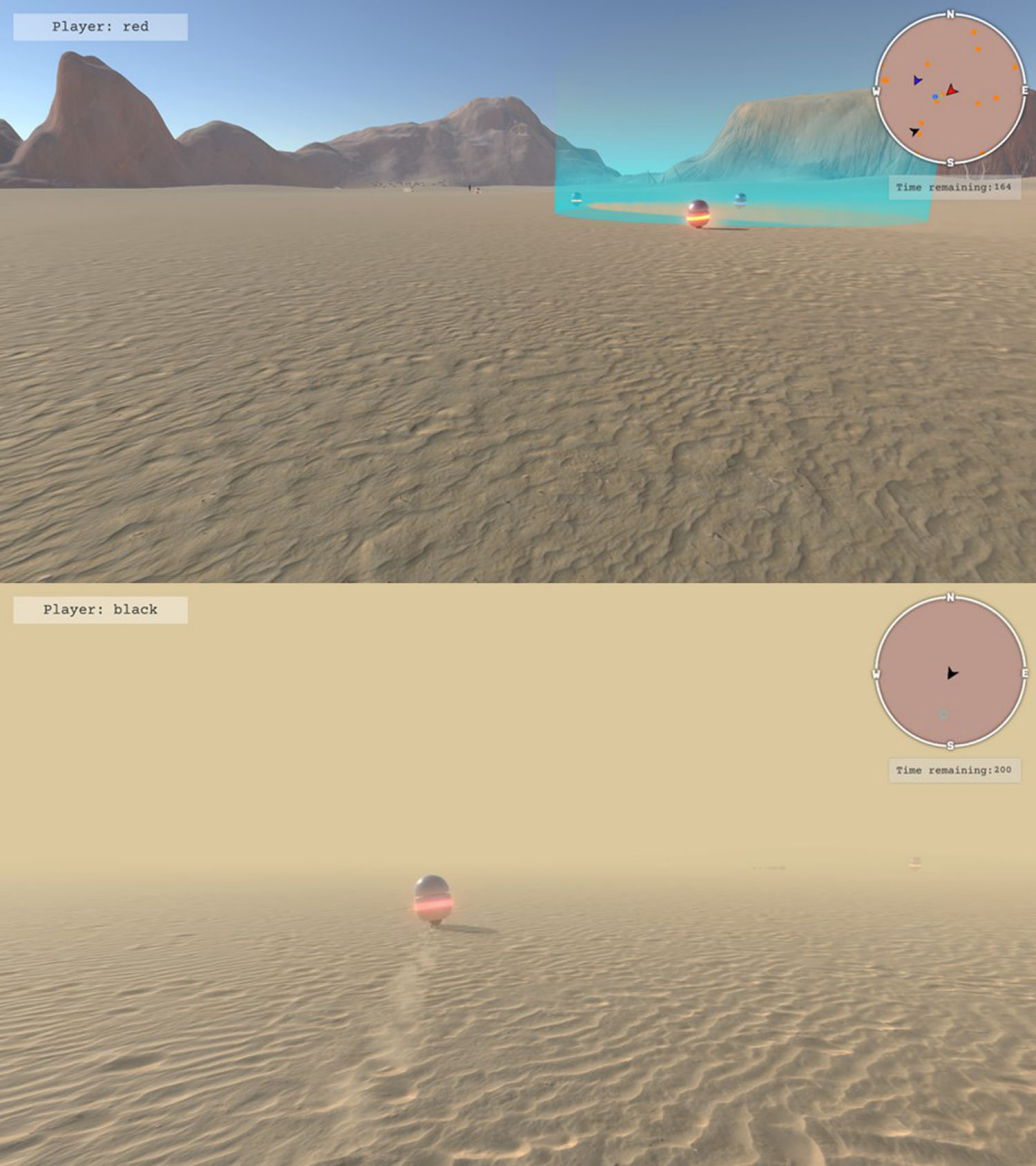
Player view of task manipulations. Participants used keyboard and mouse controls to navigate their humanoid avatars across a large desert environment. Teams consisting of three participants were tasked to search, corral, and contain TAs within the cyan containment area (see top). Participants’ ability to have SA of task‐relevant components was manipulated in two ways. Participants either had full visibility of the desert environment (top), or their vision was obscured by fog (bottom). Additionally, participants either had access to all task‐relevant information regarding the positions of both teammates and the TAs via a HUD (the minimap in the top right), or participants were only given their own direction of heading (the minimap in the bottom image). Regardless of condition, both minimaps displayed the location of the containment area (represented as a cyan circle). Participants were also presented with a timer indicating how much time was remaining in a trial as well as a nametag to notify of their player's name in the experiment.

**Fig. 3:**
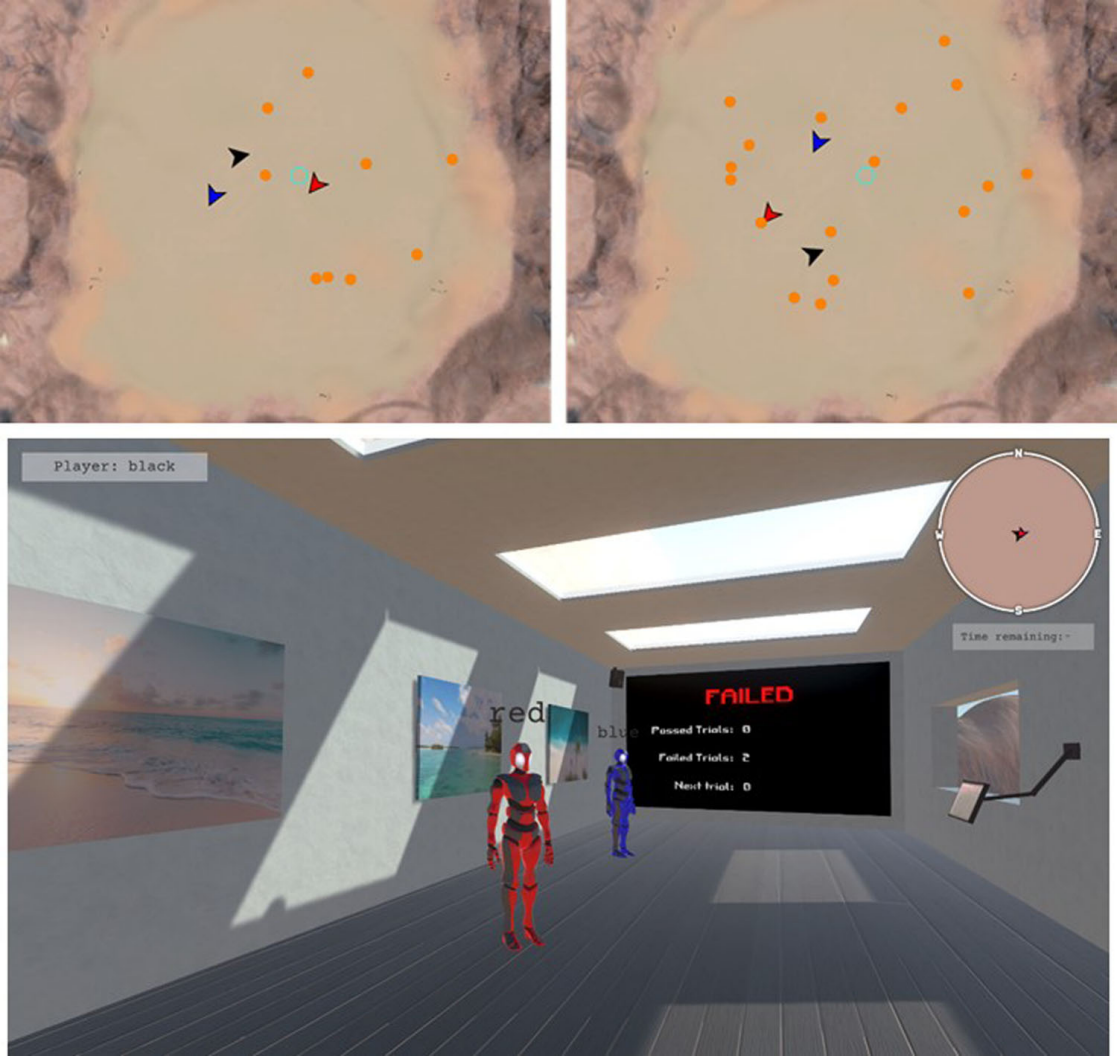
Example initial arrangement of TAs and game lobby. Three‐person teams (represented by the blue, black, and red arrows) were required to search, corral, and contain 9 (top left) or 18 (top right) TAs (orange circles) to the containment location (cyan‐colored ring). The color of any given TA indicated its status: idle (orange), fleeing (red), contained (blue), and all targets contained (green). Note that the top map views of the environment were not available to participants but were instead represented in a HUD available to players in certain task configurations (see Fig. 2). Following each trial, participants, embodied as humanoids (bottom), were teleported to a game lobby where participants received feedback regarding the previous trial, summary of how many trials were successful/unsuccessful, and a timer to indicate when the next trial was to begin.

The game involved locating, corralling, and containing a set of robots or TAs (the spherical robots in Fig. 2) who freely roamed about a large area of desert terrain (500 × 500 m in game space). The goal of the game was for the three‐person teams to work together to locate, corral, and contain the TAs within a fixed central containment area (cyan highlighted circle in Fig. 2 [top], measuring 10 m in diameter). Participants interacted with the TAs by controlling their own humanoid game avatars which were uniquely identified by their body's color (red, blue, black) (see Fig. 3, bottom). Participants could control their avatars using their mouse to control heading direction, and the “W,” “A,” “S,” and “D” keys on the keyboard to control forward, left strafe, right strafe, and backward locomotion, respectively. When moving, the avatars would move at a rate of 10 m·s^–1^. To allow for finer control, the avatar's movements can be reduced to 5 m·s^–1^ by holding down the “left shift” key.

The game was split into trials with a maximum duration of 5 min. If teams could keep all TAs contained within the containment area for 5 continuous seconds, the trial would end early, and participants would receive feedback that they have succeeded. Otherwise, if the trial time expired, the trial would end, and participants would receive feedback that they have failed. During each trial, participants had access to how much time was remaining (see Fig. 2, bottom). At the start of each session, and between each game trial, participants’ game avatars were placed into a game lobby which overlooked the game field (see Fig. 3, bottom). The game lobby contained a display screen which showed participants how many trials were completed, including the number of which resulted in success/failure. This screen also displayed whether the preceding trial led to success or failure. Between trials, participants remained in the game lobby for 10 s.

When a trial was initiated, participants’ game avatars were randomly placed within 100 m of the containment area, whereas TAs were randomly placed within 180 m of the containment area. When left unperturbed, the TAs exhibited Brownian motion dynamics, in that a random force between 0 and 60 N was applied to the TA in a random (*x*, *y*) planar direction at a rate of 1 Hz (TAs had 1 kg mass). When a participant's avatar was within 10 m of a TA, an additional force was applied in the direction directly away from the participant's avatar. This force was inversely proportional to the distance between the participant's avatar and the TA (with a maximum force of 450 N). Both the Brownian and repulsive forces were applied until the TA reached a maximum velocity of 10 m·s^–1^.

The TAs also provided visual feedback regarding their status to participants by changing the color of the ring located around the middle of their body. The ring‐light was orange when the targets were unperturbed (and hence only exhibited Brownian dynamics), red when fleeing a nearby participant avatar, blue when the TA was contained within the containment area, and green when all TAs were contained within the containment area.

#### 3.3.2 Task manipulations

Three task manipulations were employed. First, the number of targets that teams had to locate, corral, and contain was set to either 9 or 18 targets (Target Number) (see Fig. 3, top for an example of initial arrangement). Second, the visibility of the game environment was altered via the presence of game fog (Visibility). In the absence of fog, participants had near‐perfect visibility and could see >150 m within the game space (see Fig. 2, top). In contrast, when fog was present, participant visibility was restricted to being able to see clearly for approximately 10 m (see Fig. 2, bottom). Third, the level of detail regarding game state information provided to participants was manipulated via the use of a HUD. The HUD provided either local information about the cardinal heading of the participants’ own avatar (like a compass; see Fig. 3, bottom), or global information about the relative locations and headings of all participant avatars and TAs, including the TAs ring‐color “state” (Fig. 2, top). For both the compass and global HUDs, the containment area was provided to serve as a landmark.

For each session, teams completed two blocks, each consisting of eight trials representing all possible combinations of the task manipulations (Target Number, Visibility, HUD). For the first three sessions, trial order for each block was pseudorandomized. For the fourth and final session, all teams completed the same random trial order.

#### 3.3.3 Procedure

Participants who were recruited for the study were notified via e‐mail specifying their four allocated session times and asked to complete a preliminary survey in which informed consent was obtained, links to download the game software were provided, and general task and session instructions were presented. After all members of a team had completed the preliminary survey, a confirmation e‐mail was sent informing the team that everyone in their team had confirmed their participation and they were reminded of their scheduled session times. Each participant in a team was assigned a color (red, blue, or black) which served as their name for the experiment, as well as the color of their in‐game player avatar.

For each session, an e‐mail was sent 10 min prior to the scheduled meeting time containing the link to the Zoom meeting. Upon entering the Zoom meeting, participants were asked to rename their username to their assigned color and to keep their video camera turned off. Once all participants did this, the experimenter began audio recording. The experimenter's microphone was muted during game play. During the experiment, including intertrial periods, participants could freely converse with each other (see the Supporting Information for an analysis of team communication frequency in this experiment). For the experiment, an audio file for each participant was recorded as well as a combined audio stream of all participants in the team. Both the individual participants’ and teams’ combined audio files were recorded to facilitate audio transcription and verbal/conversation coding. Note that these data were not analyzed here but were recorded for the purposes of a larger study and will be analyzed in future work (see Discussion for more details).

Following audio setup, participants were asked to launch the Unity application and connect to the game server. After it was confirmed that all participants had joined the game session and that their Internet connection was stable, the game trials commenced. There was a maximum time limit of 90 min for each session, and participants were asked to complete as many of the 16 trials as they could within that 90‐min period. If a participant disconnected during a trial (due to an Internet connection error or high latency), the trial was terminated, and the participant was asked to rejoin, and the trial was redone (this occurred on 13.37% of trials). However, if a team was near trial success when a team member dropout occurred (i.e., all TAs were contained in the containment area, but for less than 5 s), the trial was not redone after the team member rejoined the game session and the trial was deemed successful.

After completing all trials or after the 90‐min session time had elapsed, the experimental session was stopped, and the Zoom call ended. This procedure was followed for all four sessions. Following the fourth and final session, participants were debriefed about the purpose of the study and thanked for their participation.

### 3.4 Measures

All completed trials (unsuccessful and successful) were included in analysis. The following measures were computed.

#### 3.4.1 Team performance

At the team level, task performance was assessed using each trial's duration in seconds. Here, lower values indicated faster trial completion times with an upper ceiling value of 300 s for failed trials, representing the maximum trial duration.

#### 3.4.2 Team division of labor

A secondary team‐level measure to assess team performance was to quantify the extent to which teams divided labor by partitioning the search space. It was expected that teams who subdivided the search space between the three team members would excel at completing the task within the allotted trial time as it would enable the parallelization of search. This contrasts with a strategy where all participants moved together to search the same regions. This division of labor was quantified as the proportion of the search area that was overlapping between two or more players. Here, 0 would indicate that teams cleanly partitioned the search space, while 1 would indicate that all participants searched the same locations.

**Fig. 4:**
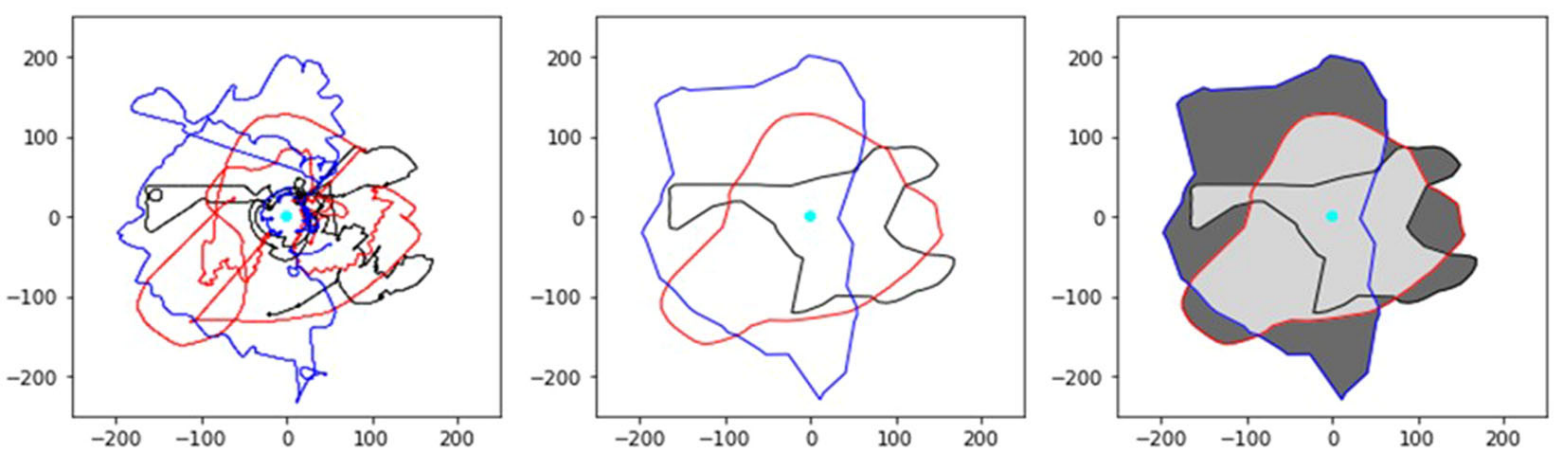
Illustration of how proportion overlap of player search was calculated. The position time series of each participant's avatar (black, red, blue) (left) is submitted to the alphashape python toolbox. Tightest‐fitting polygons for each participant are then generated (middle), which represent the participants’ search areas. Polygon “fragments” are then obtained by taking the intersection of each pair‐wise combination of participant search areas and then combined (light gray polygon in the right panel). The proportion overlap of a team's search area is then computed as the area of the light gray polygon divided by the total spread (represented as the sum of the dark gray and light gray polygons in the right panel). The cyan circle represents the location of the containment area.

For each participant, a bounding polygon which encapsulated the participant's avatar movements was created using the alphashape python toolbox (https://pypi.org/project/alphashape/) to quantify each participant's search area. The construction of the bounding polygon is an iterative process whereby a circle with radius *r* ^−1^ is “rolled” along the extremities of the dataset, producing polygon edges whenever two data points intersect the circle. This process is repeated until the tightest‐fitting single polygon is found (see Fig. 4). Failure for *r* ^–^^1^ to converge will result in the convex hull being used as the bounding polygon. The convex hull represents the smallest possible convex bounding polygon (whose shape is akin to a rubber band wrapped around pegs on a pegboard). Once the bounding polygons were created, the proportion of the search area that was overlapping was measured as the area spanning the overlapping polygons divided by the total search area. For this measure, each participant movement time series was downsampled to 5 Hz and the first second was deleted to remove any transient periods in behavior.

#### 3.4.3 Participant search behavior structure

At the individual level, the persistent structure of participants’ movement dynamics during search and containment was quantified using DFA (Hardstone et al., 2012; Peng et al., 1994), implemented by using the nolds python toolbox (https://pypi.org/project/nolds/). For this study, search behavior was measured by assessing the displacement of each participants’ position as well as their head/screen orientation. Specifically, for displacement, a time series was constructed which measured the angle of the position displacement vector between adjacent timepoints. For head orientation, a time series was constructed which measured the change in head‐orientation angle between adjacent timepoints. Search was quantified using these two methods because the keyboard and mouse controls enabled participants to independently control movement displacement and head orientation. Both participants’ movement and orientation time series were downsampled to 5 Hz, and the first second was deleted to remove any transient periods in behavior.

Once both time series were constructed, DFA was calculated as the following for each time series. First, the mean of the time series was subtracted and the cumulative sum was obtained. The time series was then detrended using polynomial best fit at increasing window sizes starting from five data points (1 s) with a 50% window overlap. Window sizes were increased by multiplying the previous window size by 1.2 until the maximum value was reached that was less than 10% of the time series total length. At each window size, the windowed time series was detrended and the standard deviation was computed and averaged across all windows. The average standard deviation was then plotted on a log–log plot against the window sizes, and a best‐fit line was determined. The slope of this best‐fit line is what is referred to as DFA_α_. Here, DFA_α_ = 0.5 indicates random fluctuations (i.e., no structure) in participant's displacement or orientation behavior across time (i.e., akin to white noise), and DFA_α_ = 1.0 corresponds to moderately persistent behavioral fluctuations (i.e., pink noise structure), whereas DFA_α_ = 1.5 reflects highly persistent behavioral structure (i.e., akin to Brownian motion).

## 4 Results

All 10 teams completed all four experimental sessions. Not all trials were completed for two of the experimental sessions: for Session 1, five teams did not complete all trials within the experiment time limit (7, 2, 3, 2, and 3 trials missing, respectively); for Session 2, one team did not complete all trials (one trial missing); for a total of 18 (2.81%) trials that were not completed out of 640 total trials. Across the completed trials, one participant disconnected due to Internet connectivity issues, resulting in partial data for 18 of the 622 recorded trials (2.89%). For trials containing partial data, only one participant experienced a disconnection. Only trials where all three participants were present for the entire duration were included in analyses.

For each dependent measure, multilevel (mixed‐effects) models were fitted. For these models, the model fitting procedure and validation results are summarized in the Supporting Information. As explained in more detail in the supplemental document, accelerated failure time (AFT) survival analysis regressions were used to model trial duration. Further, search area overlap values were Box–Cox transformed (λ = 0.46) before being modeled due to the data being non‐normally distributed. The data for the DFA_α_ measures were normally distributed and thus did not require special treatment. In the reported results, all *p* values and confidence intervals were Bonferroni corrected.

**Fig. 5:**
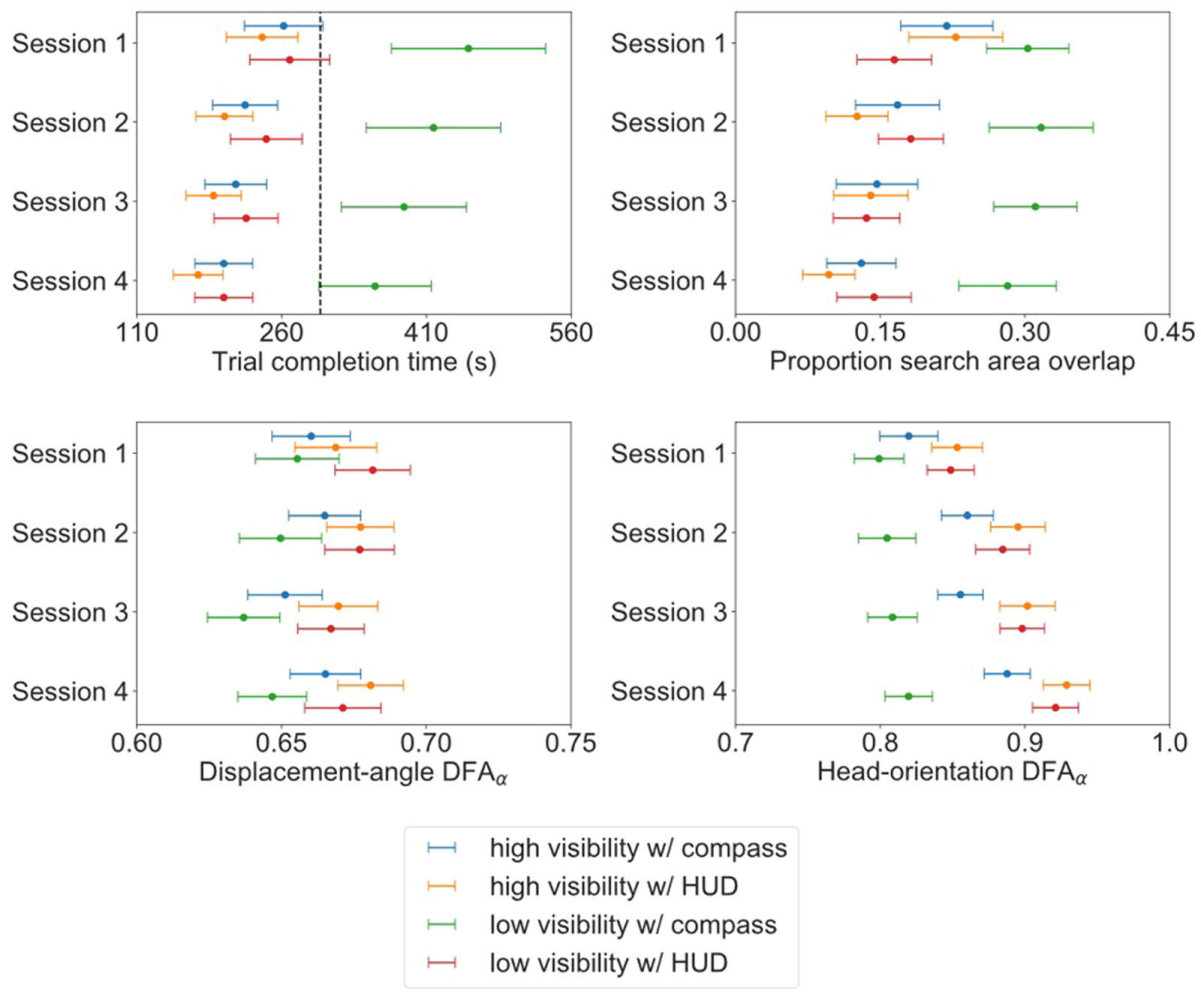
Summary of results. (Top left): Average adjusted predictions for trial completion time from the final fitted model; (top right): The sample's mean proportion search area overlap; (bottom left): The sample's mean participant displacement‐angle DFA_α_; (bottom right): The sample's mean participant head‐orientation DFA_α_. The horizontal bars represent the 95% confidence interval for the associated measure. The black dotted line displayed in the top left plot represents the maximum time teams had to complete any given trial (300 s).

In the following, significant results pertaining to Session as well as the Visibility and HUD manipulations are presented. A summary of the results is presented in Fig. 5. To simplify the document, results pertaining to Target Number, including any interactions, are summarized in the Supporting Information. The results regarding Target Number were largely trivial—teams required more time to complete trials, participants overlapped more in where they searched, and their search was more random when teams were required to corral 18, as opposed to 9 TAs. Given the random distribution of where TAs were initialized, these results indicate that teams needed more time to sufficiently search the environment to locate all 18 TAs.

### 4.1 Task performance

Teams, on average, took significantly less time to search, corral, and contain TAs when players’ vision was not obscured by fog (average adjusted prediction, *AAP* = 211.89 s, *SE* = 14.9) compared to when fog was present (*AAP* = 319.0, *SE* = 23.3; average marginal effect, *AME* = –107.1, *SE* = 10.9, 95% CI [85.7, 128.5]; *g* = –0.37, *SE* = 0.02, *Z* = 16.54, *p* < .001, where *g* is the estimate of the contrast {–1, 1} of the non‐exponentiated marginal linear predictions from the AFT model for, in this case, high visibility/low visibility). Similarly, teams took significantly less time when players had access to all task‐relevant information via the presence of the HUD (*AAP* = 216.5 s, *SE* = 15.2) compared to via a compass (*AAP* = 312.2 s, *SE* = 22.8; *AME* = –95.7, *SE* = 10.3, 95% CI [–115.8, –75.6]; *g* = –0.32, *SE* = 0.02, *Z* = –14.48, *p* < .001). Further, by the fourth session (*AAP* = 232.8 s, *SE* = 17.0), teams completed the trials significantly faster than during their first session (*AAP* = 306.1 s, *SE* = 22.9; *AME* = –73.3, *SE* = 11.1, 95% CI [–95.1, –51.5]; *g* = –0.30, *SE* = 0.03, *Z* = –9.72, *p* < .001).

In addition to the main effects, there was a significant Visibility × HUD interaction. Teams took significantly less time to complete trials when they had access to global information via the HUD, as opposed to a compass, across both low visibility (*AME* = –169.3, *SE* = 18.4, 95% CI [–205.4, –133.2]; *g* = –0.54, *SE* = 0.04, *Z* = –15.01, *p* < .001) and high visibility (*AME* = –23.3, *SE* = 5.8, 95% CI [–34.7, –11.8]; *g* = –0.11, *SE* = 0.03, *Z* = –4.16, *p* < .001) trials. However, the benefit of having access to the HUD was greater when visibility of the environment was poor (*AME* = –146.0, *SE* = 17.8, 95% CI [–181.0, –111.0]; *g* = 0.44, *SE* = 0.04; *Z* = 9.94, *p* < .001). There were no significant HUD × Session (χ2(3) = 0.91, *p* = .824) nor Visibility × Session (χ2(3) = 1.97, *p* = .579) interactions. A summary of the overall success rate for each task manipulation is presented in Supplemental Table 1.

### 4.2 Division of labor

The results above indicated that with experience, players developed effective strategies to reduce the amount of time needed to complete a trial. The availability of a HUD facilitated task performance, especially when visibility of the environment was obscured by the presence of fog. The improvements in performance with experience were expected to be due to teams devising better division of labor strategies to reduce the redundancy of search behaviors for TAs. The ability to divide labor effectively was also expected to be impacted by the ease of obtaining task‐relevant information to facilitate the development of SA.

Indeed, the search areas of participants overlapped more when visibility of the environment was poor (*M* = 0.23, *SE* = 0.01) compared to when there was clear visibility (*M* = 0.16, *SE* = 0.01; *g* = 0.21, *SE* = 0.04, *t*(9.4) = 5.957, *p* < .001, where *g* is the estimate of the contrast {–1, 1} of the marginal linear predictions from the multilevel model predicting the Box–Cox‐transformed search overlap scores, and the *t* degrees of freedom are Kenward–Roger estimates, as explained in the Supporting Information). Similarly, the search areas of participants overlapped more when participants only had access to a compass (*M* = 0.23, *SE* = 0.01) as opposed to the HUD (*M* = 0.15, *SE* = 0.01; *g* = 0.21, *SE* = 0.04, *t*(9.6) = 5.49, *p* < .001). Additionally, with experience, participants were more effective in dividing their search by the fourth session (*M* = 0.16, *SE* = 0.01), compared to their first (*M* = 0.23, *SE* = 0.01; *g* = –0.21, *SE* = 0.04, *t*(17.2) = –5.08, *p* < .001).

In addition to the main effects, significant Visibility × HUD (*t*(339.5) = –6.14, *p* < .001) and Visibility × Session (*F*(3, 203.7) = 5.56, *p* = .001) interactions were found. For the Visibility × HUD interaction, participants, when exposed to environmental fog, were significantly better at dividing the search area when they were given access to a HUD (*M* = 0.16, *SE* = 0.01) compared to a compass (*M* = 0.30, *SE* = 0.01; *g* = –0.36, *SE* = 0.05, *t*(20.8) = –7.75, *p* < .001). When participants had clear visibility of the task environment, there was no improvement in reducing search area overlap when given a HUD (*M* = 0.15, *SE* = 0.01) over a compass (*M* = 0.16, *SE* = 0.01; *g* = –0.05, *SE* = 0.04, *t*(18.1) = –1.21, *p* = .487).

For the Visibility × Session interaction, the presence of fog increased the amount of overlap in participant search areas for the second (*M_clear_* = 0.15, *SE_clear_* = 0.01; *M_fog_* = 0.25, *SE_fog_* = 0.02; *g* = 0.28, *SE* = 0.06, *t*(45.2) = 5.03, *p* < .001), third (*M_clear_* = 0.14, *SE_clear_* = 0.01; *M_fog_* = 0.22, *SE_fog_* = 0.02; *g* = 0.23, *SE* = 0.05, *t*(44.6) = 4.15, *p* < .001), and fourth and final session (*M_clear_* = 0.11, *SE_clear_* = 0.01; *M_fog_* = 0.21, *SE_fog_* = 0.02; *g* = 0.28, *SE* = 0.06, *t*(30.9) = 4.67, *p* < .001), with no difference in the Visibility manipulation in the first session (*M_clear_* = 0.22, *SE_clear_* = 0.02; *M_fog_* = 0.23, *SE_fog_* = 0.02; *g* = 0.03, *SE* = 0.06, *t*(50.0) = 0.59, *p* > .999). Further, the overlap in participant search areas did not differ between the four sessions when there was fog (*F*(3, 78.6) = 1.31, *p* = .278) but did when participants had a clear view of the environment (*F*(3, 86.2) = 16.01, *p* < .001). Specifically, with experience (from the first to fourth session), participants reduced the amount of search area that was overlapping when they had full visibility of the environment (*g* = –0.34, *SE* = 0.05, *t*(41.9) = –6.49, *p* < .001), but did not do so when fog was present (*g* = –0.09, *SE* = 0.06, *t*(34.5) = –1.49, *p* = .293). There was no significant HUD × Session interaction (*F*(3, 171.4) = 0.24, *p* = .867).

**Fig. 6:**
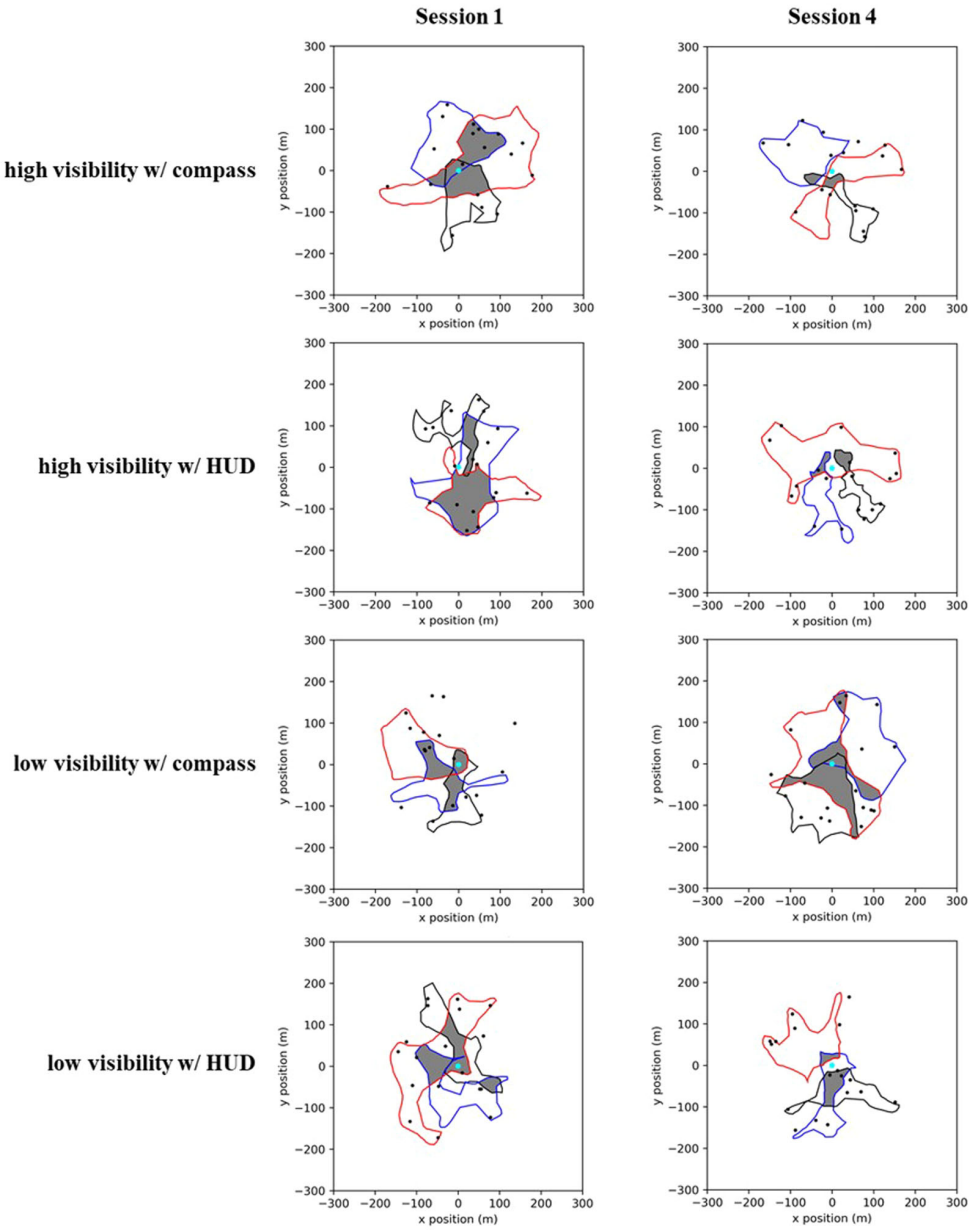
Example team search area overlaps. Examples of participant search areas and their overlap (in gray), from the same team, as a function of experience and task manipulations. Each colored polygon represents the tightest‐fitting polygon that contains each player's movement trajectory during the trial. The initial positions of the TAs are also shown as black circles (only the 18 TA condition is shown). With experience, and when participants either had clear visibility of the environment or a HUD that displayed the positions of all TAs and their team members, teams learned to reduce the redundancy for where they searched for TAs.

Like the results regarding task performance, teams were similarly impacted by the manipulations in how they were able to effectively divide their search. With experience, teams learned to coordinate and divide their search activities more effectively. Indeed, reductions in search area overlap were associated with faster task completion (see Supplemental Figure 3). This coordination was facilitated by factors that enabled participants to obtain SA of the locations of their teammates (e.g., clear visibility, HUD). When participants were impacted by fog, however, the ability to develop more efficient division of labor strategies was hampered. (See Fig. 6 for examples of search areas from the same team when completing the task in the different experimental manipulations.)

### 4.3 Search behavior structure

The results regarding task performance and search division of labor indicate that teams, with experience, learned to develop effective strategies to solve the task more efficiently. This development was facilitated by assistive technologies like a HUD or by having unobstructed vision of the task environment which contributes to the development of SA of the task context. To further determine whether the development of SA to facilitate task performance was embodied by participants, the structure of participant search behaviors was assessed using DFA. For each trial, DFA_α_ was computed on both the time series of the directions of the displacement vector (displacement angle) as well as the angular change of the participant head orientation (head orientation), as both could be independently controlled by participants.

#### 4.3.1 Analysis of displacement‐angle DFA_α_

Participants structured their movement displacements in a more prospective manner when there was relative ease in obtaining task‐relevant information. Specifically, participants had greater displacement‐angle DFA_α_ values when they had access to the HUD (*M* = 0.674, *SE* = 0.002) compared to when only having access to a compass (*M* = 0.654, *SE* = 0.002; *g* = 0.020, *SE* = 0.004, *t*(10.08) = 4.73, *p* < .001, where *g* is the estimate of the contrast {–1, 1} of the marginal linear predictions in the displacement‐angle DFA_α_ multilevel model). While on average, there was no significant difference in displacement‐angle DFA_α_ values between trials when visibility was clear (*M* = 0.667, *SE* = 0.002) compared to when there was fog (*M* = 0.660, *SE* = 0.002; *g* = 0.006, *SE* = 0.003, *t*(33.6) = 2.01, *p* = .052), there was a significant HUD × Visibility interaction (*g* = 0.013, *SE* = 0.005, *t*(59.7) = 25.54, *p* = .014). When participants had access to the compass, their movement displacements were less structured when their vision was obscured by fog (*M* = 0.647, *SE* = 0.003) than when they had clear visibility (*M* = 0.661, *SE* = 0.003; *g* = –0.013, *SE* = 0.004, *t*(28.9) = –2.83, *p* = .001). However, when participants were given access to a HUD, the structure of their movement displacements was not impacted by the visibility of the environment (*M_clear_* = 0.674, *SE_clear_* = 0.003, *M_fog_* = 0.674, *SE_clear_* = 0.003; *g* < 0.001, *SE* = 0.003, *t*(67.8) = 0.18, *p* > .999).

Examining the HUD × Visibility interaction from the other direction, the presence of the HUD resulted in participants exhibiting greater structure in their movement displacements in both clear visibility (*g* = 0.013, *SE* = 0.005, *t*(13.2) = 2.99, *p* = .021) and fog trials (*g* = 0.026, *SE* = 0.005, *t*(19.8) = 4.89, *p* < .001). However, the impact of the HUD was greater when participants’ vision was obscured by fog (*g* = 0.013, *SE* = 0.005, *t*(59.7) = 2.54, *p* = .014). There were no significant differences in displacement‐angle DFA_α_ values between sessions (*F*(3, 26.8) = 0.42, *p* = .741), and there was no significant HUD × Session (*F*(3, 41.7) = 0.59, *p* = .627) interaction. Although there was a significant Visibility × Session (*F*(3, 434.5) = 2.83, *p* = .038) interaction, there was no significant effect of the visibility manipulation across sessions (all *t* ≤ 2.48, *p* ≥ .06).

#### 4.3.2 Analysis of head‐orientation DFA_α_

Participants exhibited more structured scanning behaviors, assessed by their head rotations, when there was relative ease in obtaining task‐relevant information. Further, players became more structured in how they scanned the environment as a function of experience. Specifically, participants had greater head‐orientation DFA_α_ values if they had a clear view of the environment (*M* = 0.876, *SE* = 0.003) compared to when there was fog (*M* = 0.848, *SE* = 0.003; *g* = 0.027, *SE* = 0.012, *t*(13.1) = 2.25, *p* = .042, where *g* is the estimate of the contrast {–1, 1} of the marginal linear predictions in the head‐orientation DFA_α_ multilevel model). Similarly, participants had greater head‐orientation DFA_α_ values when global information was presented via a HUD (*M* = 0.892, *SE* = 0.003) compared to via a compass (*M* = 0.833, *SE* = 0.003; *g* = 0.059, *SE* = 0.007, *t*(13.2) = 7.77, *p* < .001). Further, participants had greater head‐orientation DFA_α_ values in the fourth session (*M* = 0.889, *SE* = 0.005) compared to the first session (*M* = 0.830, *SE* = 0.005; *g* = 0.056, *SE* = 0.012, *t*(9.2) = 4.82, *p* = .003). Head‐orientation DFA_α_ values did not differ between the first and second (*M* = 0.861, *SE* = 0.005; *g* = 0.028, *SE* = 0.011, *t*(9.4) = 2.57, *p* = .088) and first and third sessions (*M* = 0.866, *SE* = 0.005; *g* = 0.032, *SE* = 0.013, *t*(9.2) = 2.56, *p* = .091).

In addition to the main effects, there were significant Visibility × HUD (*t*(9.9) = 3.17, *p* = .010), Visibility × Session (*F*(3, 40.4) = 3.59, *p* = .021), and HUD × Session (*F*(3, 111.7) = 5.75, *p* = .001) two‐way interactions. Additionally, the three‐way Visibility × HUD × Session interaction was also significant (*F*(3, 254.8) = 3.27, *p* = .022).

Decomposing the Visibility × HUD interaction, head scanning behaviors were less structured when participants only had access to a compass, compared to the HUD, both when visibility was (*M_compass_* = 0.808, *SE_compass_* = 0.005, *M_HUD_* = 0.889, *SE_HUD_* = 0.004; *g* = –0.080, *SE* = 0.013, *t*(10.0) = –6.08, *p* < .001) and was not (*M_compass_* = 0.857, *SE_compass_* = 0.005, *M_HUD_* = 0.896, *SE_HUD_* = 0.005; *g* = –0.039, *SE* = 0.005, *t*(10.3) = –7.10, *p* < .001) obscured by fog. However, this difference was larger when vision was obscured by fog (*g* = 0.041, *SE* = 0.013, *t*(9.9) = 3.17, *p* = .010).

Decomposing the Visibility × Session and HUD × Session interactions demonstrates that head‐orientation DFA_α_ values increased from the first to the fourth session regardless of the manipulation. Specifically, participants were more structured in their scanning behaviors with experience (i.e., between Sessions 1 and 4) both when there was (*M_S1_* = 0.824, *SE_S1_* = 0.006, *M_S4_* = 0.870, *SE_S4_* = 0.007; *g* = 0.044, *SE* = 0.012, *t*(11.2) = 3.55, *p* = .009) and was not (*M_S1_* = 0.836, *SE_S1_* = 0.007, *M_S4_* = 0.908, *SE_S4_* = 0.006; *g* = 0.068, *SE* = 0.012, *t*(11.1) = 5.59, *p* < .001) fog present. However, this improvement was greater when the environment had no fog (*g* = 0.024, *SE* = 0.008, *t*(23.5) = 3.04, *p* = .006). Similarly, participants were more structured in their scanning behaviors with experience both when they had (*M_S1_* = 0.851, *SE_S1_* = 0.006, *M_S4_* = 0.925, *SE_S4_* = 0.006; *g* = 0.071, *SE* = 0.012, *t*(11.4) = 5.80, *p* < .001) and did not have (*M_S1_* = 0.810, *SE_S1_* = 0.007, *M_S4_* = 0.855, *SE_S4_* = 0.006; *g* = 0.041, *SE* = 0.012, *t*(11.3) = 3.34, *p* = 0.13) access to the HUD. However, this improvement was greater for trials where the HUD was available (*g* = 0.030, *SE* = 0.008, *t*(1464.5) = 3.88, *p* < .001).

When decomposing the Visibility × HUD × Session three‐way interaction, the Visibility × HUD effects described above was found to only be significant for the second (*t*(21.4) = 2.76, *p* = .046), third (*t*(21.5) = 2.78, *p* = .045), and fourth and final (*t*(21.8) = 3.89, *p* = .003) sessions (first session: *t*(23.8) = 0.87, *p* > .999). Further, the Visibility × Session two‐way interaction described above was significant only across trials where participants only had access to a compass (*g* = 0.035, *SE* = 0.011, *t*(657.12) = 3.18, *p* = .008). The interaction was not significant when participants had global information in the HUD (*g* = 0.005, *SE* = 0.011, *t*(763.3) = 0.49, *p* > .999). Finally, the HUD × Session two‐way interaction (comparing Sessions 1 and 4) described above was marginally significant when fog was present in the environment (*g* = 0.031, *SE* = 0.012, *t*(169.5) = 2.57, *p* = .066), but not when there was clear visibility of the environment (*g* = 0.001, *SE* = 0.012, *t*(161.88) = 0.10, *p* > .999).

In summary, the DFA analyses investigating both the structure of how participants moved across the task environment and scanned their environments demonstrated the following. For displacement‐angle DFA_α_, having access to a HUD or clear visibility of the environment increased the structure of how participants planned their movements across the task environment by giving participants relative ease in developing SA of the task environment and the location of other players. When investigating the scanning behaviors of participants, head‐orientation DFA_α_ decreased (and thus, participants’ scanning behaviors were less structured) when obtaining task‐relevant information was difficult. Specifically, head scanning behaviors were less structured in the low visibility condition and when there was only access to the compass. Having access to the HUD or having a clear view of the environment increased the structure of players’ scanning behaviors, which also improved with experience.

### 4.4 The relationship between individual search behaviors and team performance

**Fig. 7:**
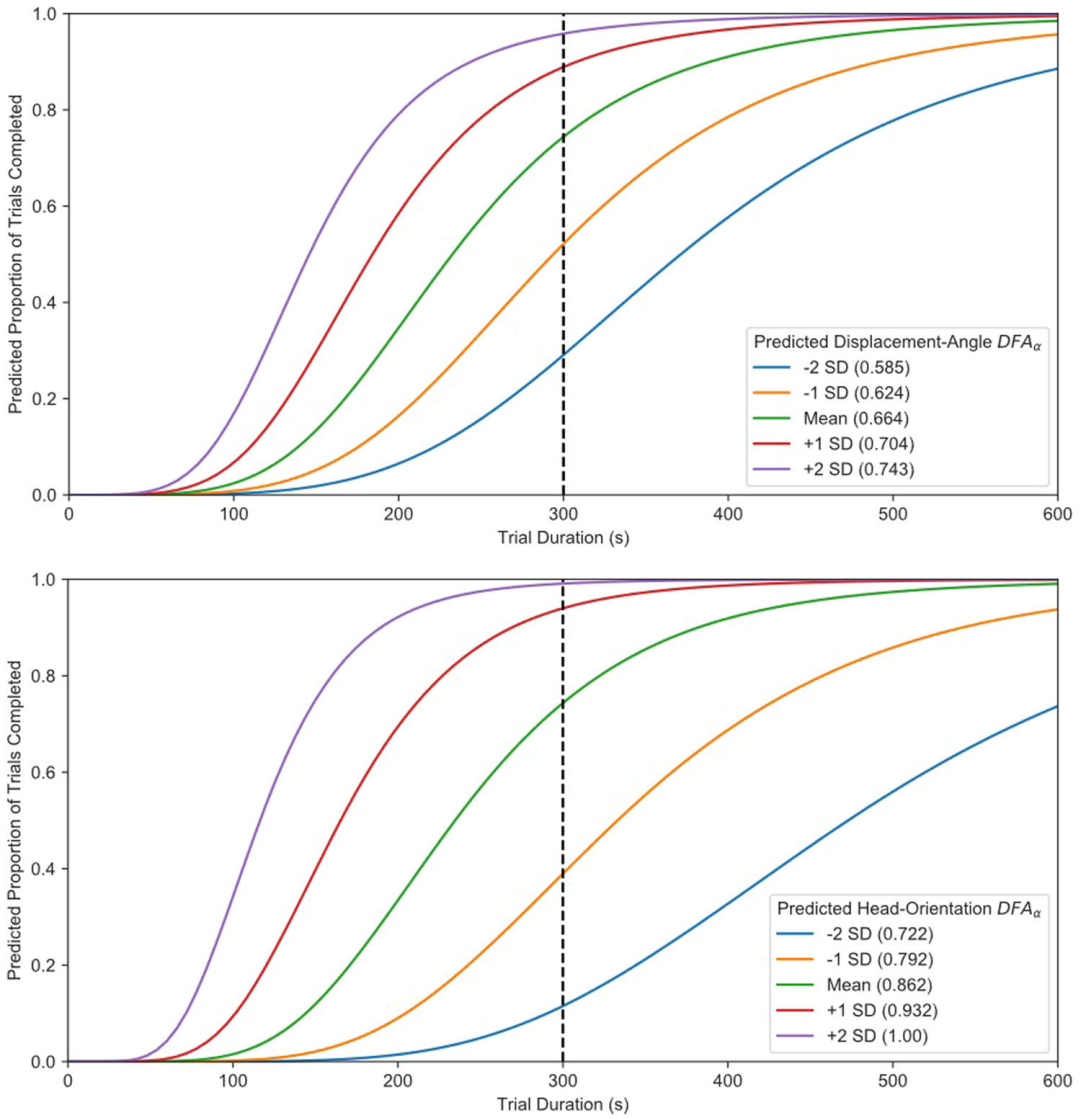
Estimated AFT survival curves including DFA_α_ as a predictor. Separate survival curve estimates are plotted for displacement‐angle DFA_α_ (top) and head‐orientation DFA_α_ (bottom). The *y*‐axis represents the proportion of teams who were expected to have completed the trial by the time interval. Separate survival curves are plotted representing DFA_α_ values that are –2, –1, 0, 1, and 2 standard deviations above the mean DFA_α_ value—with their associated values in parentheses. As can be seen with both measures, teams who were expected to have greater DFA_α_ values were predicted to complete a trial in less time. The vertical dotted black lines represent the total time teams had to complete any given trial (300 s).

Players who possess SA of the task context appeared to be able to structure their behaviors in conjunction with their team members to behave more efficiently. In the experiment employed here, efficiency was defined as how rapidly teams could search, corral, and contain the TAs within the containment region. To determine whether the development of SA was related to how players structured their behaviors during on‐going task performance, log‐logistic AFT survival analyses were conducted which included either the fixed‐effect of displacement‐angle DFA_α_ or head‐orientation DFA_α_ to predict trial completion times, with the random effect of team number (see Supporting Information's Trial Duration heading for more information). The estimated survival curves are presented in Fig. 7.

The displacement‐angle DFA_α_ model fit the data more appropriately than the base log‐logistic model (LR χ2(1) = 143.38, *p* < .001, ΔAIC = –146.38, where AIC stands for the Akaike information criterion) and indicated that team members with greater displacement‐angle DFA_α_ values took significantly less time to complete the task (*b* = –5.90, *SE* = 0.45, *Z* = –13.04, *p* < .001, where *b* is the non‐exponentiated displacement‐angle DFA_α_ coefficient in the model). Similarly, the head‐orientation DFA_α_ model fit the data more appropriately than the base log‐logistic model (LR χ2(1) = 437.20, *p* < .001, ΔAIC = –435.20) and indicated that team members with greater head‐orientation DFA_α_ values also took significantly less time to complete the task (*b* = –5.01, *SE* = 0.22, *Z* = –23.34, *p* < .001).

## 5 Discussion

The study explored whether the ability of three‐person teams to develop effective division‐of‐labor strategies during a collaborative search and retrieval task could be quantified by how team members structured their individual search behaviors using DFA. Variation in DFA's output, DFA_α_, was expected to predict the extent to which participants possess the necessary SA to prospectively control and plan their own movements in concert with others. The manipulations introduced in this study varied the ability of team members to develop SA and impacted DFA_α_ in the directions consistent with theory. Specifically, when exposed to poor visibility and when participants only had access to a compass, DFA_α_ decreased, indicating less persistence in how participants structured their search behaviors. Conversely, when participants had good visibility of the task environment, or when participants were provided with global information of all task‐relevant components, DFA_α_ increased, reflecting a stronger persistence in the behavioral time series indicating that participants were better able to prospectively control their search behaviors. Additionally, head‐orientation DFA_α_ increased as a function of experience, demonstrating that participants learned to develop scanning strategies that enabled participants to behave with more certainty. Finally, the results demonstrated that variation in DFA_α_ of team member search behaviors can predict how effective teams are in completing the task.

Although the focus of the analyses was regarding how displacement‐angle and head‐orientation DFA_α_ values were impacted by various task constraints which were hypothesized to impact team members’ ability to develop SA, it is interesting to note that the distributions for the two DFA_α_ measures were different (see Supplemental Figure 4). Specifically, the distribution of displacement‐angle DFA_α_ values (*M* = 0.66, *SD* = 0.07) was lower than the values for the head‐orientation DFA_α_ measure (*M* = 0.86, *SD* = 0.10). This may be due to the structure of participant displacement behaviors reflecting the random placement of TAs across the task environment (Humphries et al., 2010), which was further impacted when the number of TAs increased (see the Supporting Information). Future research could explore collaborative search strategies in more naturalistic settings where the distribution of items tends to cluster in clumps, such as foraging for food (Hills et al., 2013).

### 5.1 Measuring team situation awareness

Within the teams literature, measuring team cognition and coordination has been explored by submitting behavior (e.g., communication, actions) or neurophysiological signals to recurrence‐based quantification (Demir et al., 2017; Gorman et al., 2020; Marwan, Carmen Romano, Thiel, & Kurths, 2007) or entropy‐based analyses (Stevens & Galloway, 2019). The former serves to measure coupling strength and complexity between team members, while the latter can be used to identify challenge points in team coordination processes. The focus of this work from the past 10 years relates to not only measuring baseline measures but predicting when changes to team cognition occur, due to a change in the task that teams must perform (Wiltshire, Butner, & Fiore, 2018) or due to a coordination breakdown (Gorman et al., 2020). More recent research has started to investigate the potential for artificial agents as “synthetic teammates” to augment team functioning (Demir et al., 2017; McNeese et al., 2021; Nalepka, Gregory‐Dunsmore, Simpson, Patil, & Richardson et al., 2021) by assessing their inclusion on these behavioral and neurophysiological signals, as well as devising appropriate feedback methods to help steer teams towards desirable coordinative behaviors (Wiltshire, Steffensen, & Likens, 2020).

The use of DFA in this study provides another measure to quantify SA in team contexts. Past research examining whether individuals or teams possess SA or TSA has been done by either querying team members’ knowledge of task components and their anticipated dynamics or observing how teams adapt to “roadblocks” presented during an exercise or training (Gorman et al., 2006, 2020). The former approach assumes that team coordination requires that coacting individuals have a shared knowledge structure, while the latter approach leverages concepts from dynamic systems theory to define TSA as a team establishing a stronger synergy or stable mode of behavioral order that is robust to perturbations (Cooke, Gorman, Myers, & Duran, 2013; Gorman et al., 2006; Riley, Richardson, Shockley, & Ramenzoni, 2011). However, these approaches necessitate perturbing the ongoing interactions which limit its ability to quantify SA and TSA in situ. Instead, here SA was assessed “in the background” by analyzing the fluctuations of behavior within 5‐min trials. Although measuring TSA by assessing teams’ responses to perturbations may be possible in situ, this requires methods to quantify whether a perturbation or anomaly in team coordination dynamics is occurring. Recent research has begun to investigate such methods, such as deviation in communication dynamics from predictions made using time series forecasting algorithms (Gorman et al., 2020). Changes in the structure of time series data, quantified using DFA, could be another method to detect anomalies. Further, DFA can be applied to not only time series consisting of position displacement or head scanning behaviors, as was done here, but to any behavioral or physiological signal assuming sufficient data can be collected.

Although the task manipulations employed here were hypothesized to impact team members’ ability to develop SA (i.e., by manipulating the ease to which task‐relevant information could be obtained), a limitation of the current study is that our results were not compared to more traditional measures of SA for validation. Further, DFA, as employed here, is an individual‐level measure of behavior and thus can only assess SA, as opposed to TSA. A potential extension is to relate TSA to the covariation of DFA_α_ between team members, which has been found to occur during two‐person tasks (Abney et al., 2014; Almurad et al., 2017; Rigoli, Lorenz, et al., 2020). This observation, referred to as “complexity matching” is hypothesized to enable maximal information transmission between individuals (West et al., 2008), and thus enhance coordination. Despite these limitations, teams in the current experiment learned to develop coordination patterns which limited the redundancy in where team members searched, with reductions in search area overlap resulting in better task performance (see Supplemental Figure 3). This division of labor was further facilitated when team members could observe the environment, or when their perceptions were enhanced by the addition of a HUD. Thus, these results would imply that participants learned to develop the necessary SA required to work effectively with their teammates.

### 5.2 Team communication and role emergence

Although the use of HUDs may facilitate task performance, the role verbal communication has in facilitating team coordinative performance in these augmented environments is less known. In this experiment, the presence of a HUD, which specified the locations of team members and the TAs, resulted in a reduction in the overall amount of verbal communication exhibited by teams (see the Supporting Information). This was especially the case when team members’ views were obscured by the presence of fog. Although they can provide useful information to help establish SA, assistive technologies like HUDs may have the unintended consequence of making teams overreliant on their presence at the expense of developing adaptive team coordination and communication patterns. Future work should monitor the behaviors of team members in these augmented settings and design training methodologies to enable effective use of assistive technologies while also maintaining resilience in case of technological failure (Gorman et al., 2020; Wiltshire et al., 2020).

For trials where teams did not have access to the HUD when environmental fog was present, a small number of teams were observed to adopt different roles to facilitate the development of TSA. Specifically, when the task required the search and retrieval of 18 TAs with limited access to information (i.e., low visibility and no access to global task‐relevant information), some teams were observed to divide into two distinct roles once only one or TAs remained to be found—a “retriever” and a “guard.” Retrievers explored and retrieved the remaining TAs while the guard maintained the containment of any corralled agents. The guard also served the function of counting the number of contained TAs and communicated that information to the retrievers to inform how many TAs remained to be found. Future work will explore the communication between team members in greater detail to determine what communication patterns best enabled effective division of labor and the emergence of roles.

## 6 Conclusion

There has been a rise in interest within the cognitive science community to use video games to study cognition (see, e.g., the recent COGSCI 2021 workshop “Using Games to Understand Intelligence”; Brändle, Allen, Tenenbaum, & Schulz, 2021). Indeed, in the artificial intelligence community, video games are often used as the benchmark to rate the capabilities of agents trained using deep reinforcement learning (Mnih et al., 2015; Schrittwieser et al., 2020). Within team settings, first‐person video games provide an opportunity to investigate team communication and coordination processes with the inclusion of assistive technologies (e.g., HUDs). These virtual environments enable scenario‐based training to facilitate the development of teamwork, in addition to taskwork skills (Fiore & Wiltshire, 2016; Mathieu, Heffner, Goodwin, Salas, & Cannon‐Bowers, 2000). The identification of time‐dependent measures that can monitor team coordinative processes can enable prediction for coordination breakdowns and identify time points to facilitate team reflection (Gorman et al., 2020), as well as an opportunity to augment and enhance team performance with adaptive assistive technologies like HUDs, but also artificial agents who can be incorporated as synthetic teammates to steer team behavior (Nalepka, Gregory‐Dunsmore, et al., 2021; O'Neill, McNeese, Barron, & Schelble, 2022).

## Data availability

The data used for the analyses presented in this paper, and their associated Stata analysis scripts, can be publicly accessed at Open Science Framework https://osf.io/zhb2d/. For each trial, a plot summarizing each participant's displacement as well as their team's search area is also included.

## Supporting information

Supplemental Table 1. Overall success rate for each task manipulation.

Supplemental Figure 1. Histogram of trial duration for all trials.

Supplemental Figure 2. Histogram of proportion player search area overlap for all trials.

Supplemental Figure 3. Estimated accelerated failure time (AFT) survival curves including proportion search area overlap as a predictor.

Supplemental Figure 4. Histogram of DFA_α_ values for displacement‐angle and head‐orientation for all trials.

Supplemental Figure 5. Histogram of team word count rate for all trials.

Supplemental Tables. Final Models.
