# Supplementary material for: Assessing Team Effectiveness by How Players Structure Their Search in a First‐Person Multiplayer Video Game
Source: Cogn Sci. 2022 Oct 17;46(10):e13204. doi: 10.1111/cogs.13204 (PMC9787020; doi:10.1111/cogs.13204)
Supplement: Supplementary file 1 — Supplemental Table 1. Overall success rate for each task manipulation. [file COGS-46-e13204-s003.docx]

# Supplemental Information:

# Model Construction and Results Involving Target Number and Verbal Communication

## Multilevel Model Fitting

The multilevel (mixed-effects) models were fitted following the recommendations by Barr et al. (2013) and Meteyard and Davies (2020). Specifically, models were constructed in three stages. First, we identified the maximal random-effects structure for each model by starting with a model that included random intercepts, random slopes, and the full unstructured covariance matrix, before removing model parameters iteratively as required until the model could achieve convergence. Second, we defined the fixed-effects structure by iteratively adding groups of parameters to the model and assessing whether those additions significantly improved the fit of the model using likelihood-ratio (LR) tests. First, all one-way main-effect parameters were added simultaneously to the base model with only random effects, then additionally all the two-way, three-way, and four-way interaction parameters, respectively. When a group of parameters was rejected (i.e., when an LR test was non-significant), we inspected the contrasts of the marginal linear predictors in the larger model to ensure that no contrasts were significant and hence no parameters were being incorrectly rejected. If a contrast was significant, the parameters corresponding to that contrast were added to the smaller model and another LR test was conducted. If that test was significant, the parameters added were accepted in the model. If the LR test was non-significant, they were not accepted.

Finally, after the full model structure had been defined, we re-fit the model using restricted maximum likelihood (REML) so that Kenward-Roger estimates of degrees of freedom for the fixed effects could be calculated (thereby facilitating *t* and *F* tests of the fixed effects) (Kenward & Roger, 1997). It is important to note that Stata can only use ML when fitting survival-analysis multilevel models. Consequently, for models predicting Trial Duration, Kenward-Roger estimates of degrees of freedom could not be estimated, and all test of the fixed effects in those models were *Z* and χ^2^ tests instead. Our model fitting procedure as well as all code used to fit the models are documented in the included Stata .do files, accessible at <https://osf.io/zhb2d/>. Final models are summarized in **Supplemental File: Final Models**. All presented confidence intervals and *p* values were Bonferroni corrected.

## Trial Duration

For all eligible trials, task performance was quantified as the time needed by teams to complete a trial – where shorter trial completion time equated to better task performance. Of all eligible trials (604 in total), 195 (32.3%) of trials resulted in failure (i.e., teams could not contain the target agents [*TA*s] within the 5-minute trial time; see **Supplemental Table 1** for success rates for each condition). Given the resultant right censoring of the completion time data distribution (see **Supplemental Figure 1**), accelerated failure time (*AFT*) survival analyses were conducted. Here, ‘failure’ according to the analysis was defined as successfully completing the trial. A multilevel (mixed-effects) modeling approach was used such that in each AFT model defined in the analysis, trials (Level 1) were nested under participant teams (Level 2). Random intercepts were specified in the models, but random slopes were not as the analysis software, Stata MP (2-core) 17.0, either encountered non-continuous regions or could not refine the starting values for a multilevel survival model fit when random slopes were specified.

| **Supplemental Table 1** | | | | | |
| --- | --- | --- | --- | --- | --- |
| *Overall Success Rate for each Task Manipulation* | | | | | |
|  |  | Visibility | | | |
|  |  | Clear | | Fog | |
| Session | Target Number | Compass | HUD | Compass | HUD |
| 1 | 9 | 76.47% | 78.95% | 29.41% | 83.33% |
| 2 | 9 | 85.00% | 100.00% | 30.00% | 90.00% |
| 3 | 9 | 100.00% | 100.00% | 55.00% | 100.00% |
| 4 | 9 | 100.00% | 100.00% | 61.11% | 100.00% |
| 1 | 18 | 52.63% | 50.00% | 5.56% | 50.00% |
| 2 | 18 | 75.00% | 80.00% | 10.53% | 66.67% |
| 3 | 18 | 66.67% | 77.78% | 5.26% | 70.00% |
| 4 | 18 | 75.00% | 94.74% | 15.00% | 68.42% |

| 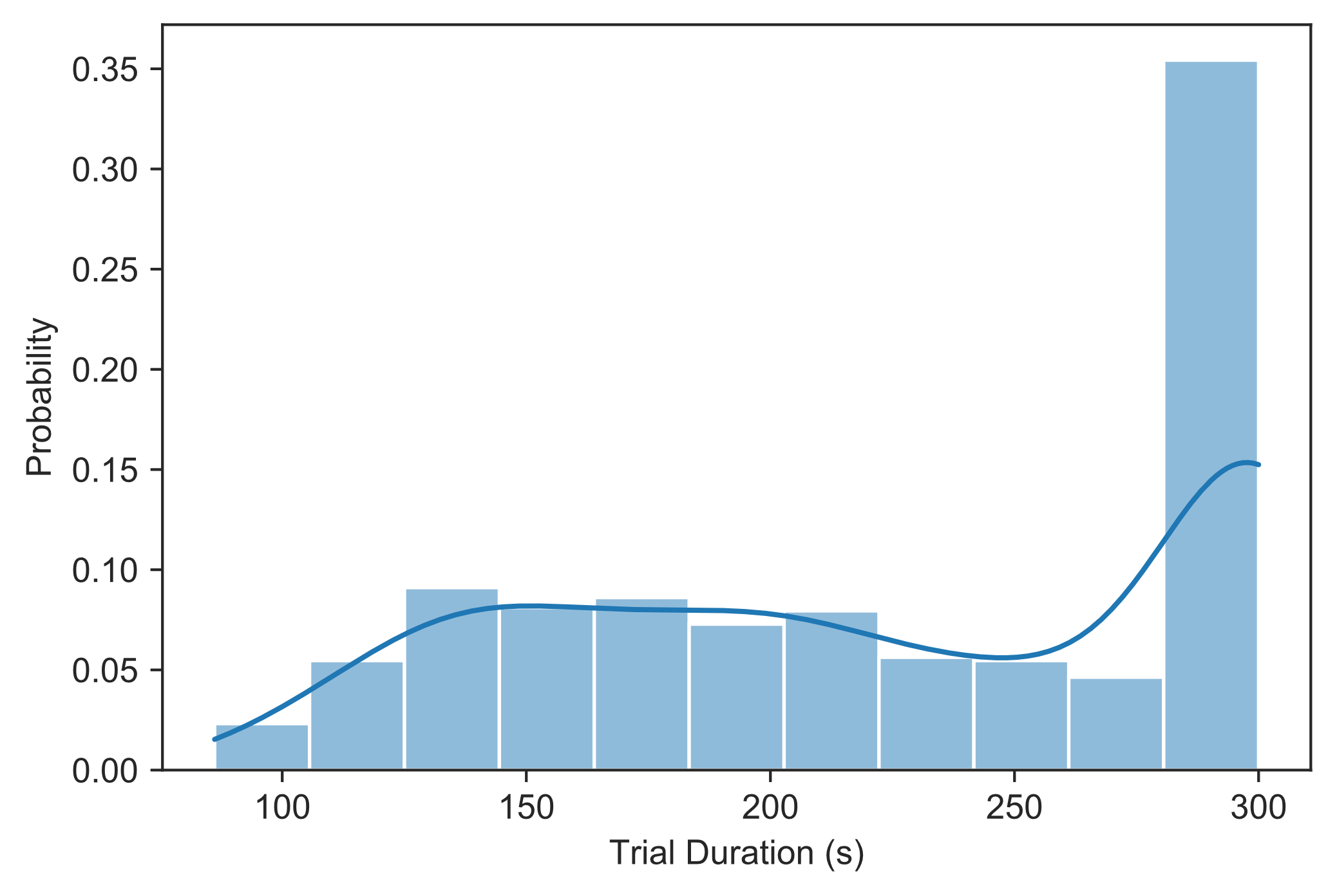  **Supplemental Figure 1. Histogram of trial duration for all trials.** Also plotted is the kernel density estimate of the data distribution (*M* = 225.00 s, *SD* = 67.70). |
| --- |

The base random-intercepts AFT models (that is, the models without any experimental-condition predictors specified) specifying exponential, Weibull, and log-logistic distributions all fit the data and better than their respective null single-level models ($\bar{\chi}^{2}$(1) = 35.58, $\bar{\chi}^{2}$(1) = 85.01, and $\bar{\chi}^{2}$(1) = 88.34 for the three likelihood ratio (*LR*) tests, respectively, and all *p* < .001). However, the log-logistic model (*LL* = -2534.37, *AIC* = 5074.74) fit the data better than the exponential (*LL* = -2765.85, *AIC* = 5535.69) and Weibull (*LL* = -2559.17, *AIC* = 5124.34) models. This suggested that the underlying hazard function for the task was non-monotonic, and hence the log-logistic distribution was preferred for all AFT models used for analyzing task performance.

Adding the fixed effects *Target Number*, *Visibility*, *HUD*, and *Session* to the base log-logistic model improved how well it fit the data (LR *χ^2^*(6) = 571.59, *p* < .001, Δ*AIC* = -559.59), as did additionally adding the two-way-interaction fixed effects (LR *χ^2^*(12) = 121.79, *p* < .001, Δ*AIC* = -97.79). However, additionally adding the three-way-interaction fixed effects did not improve the two-way model’s fit of the data (LR *χ^2^*(10) = 10.48, *p* = .400, Δ*AIC* = 9.95). Consequently, the two-way model was accepted as the most appropriate fit for the data.

To assess the significance and size of the experimental-condition fixed effects, Wald tests of the fixed-effect contrasts were conducted. In addition to the results presented the main text, Wald tests of the fixed effects in the two-way model were significant for the *Target Number* main effect (*Z* = 19.64, *p* < .001) and the *Target Number × Session* interaction (*χ^2^*(3) = 12.44, *p* = .006). The following effects were not significant: *Target Number × HUD* (*Z* = -1.09, *p* = .276) and *Target-Number × Visibility* (*Z* = -0.50, *p* = .615).

For *Target Number*, teams took significantly less time to search, corral and contain 9, (Average Adjusted Prediction, AAP = 209.6 s, SE = 14.8) as opposed to 18 (AAP = 321.0 s, SE = 23.3), TAs (Average Marginal Effect, *AME* = -111.3, *SE* = 10.7, 95% CI [-132.2, -90.4]; *g* = -0.43, *SE* = 0.02, *Z* = -19.64, *p* < .001, where *g* is the estimate of the contrast {-1, 1} of the non-exponentiated marginal linear predictions from the AFT model). For the *Target Number × Session* interaction, the improvement in team performance (i.e., faster trial completion time) from the first to the fourth session was statistically similar (*AME* = -2.6, *SE* = 17.3, 95% CI [-36.5, 31.3]; *g* = -.013, *SE* = 0.06, *Z* = -0.46, *p* = .643) for when 9 TAs had to be contained (*AME* = -74.6, *SE* = 10.5; *g* = -0.36, *SE* = 0.04, *Z* = -9.29, *p* < .001) as well as for 18 TAs (*AME* = -72.0, *SE* = 17.0; *g* = -0.23, *SE* = 0.04, *Z* = -5.09, *p* < .001).

## Division of Labor

We next explored how teams divided labor in terms of how teams partitioned the search space to locate and corral the TAs. A team’s ability to divide their search labor was defined by the degree to which search areas overlapped between two or more team members, quantified as a proportion to the total area teams searched. The *search area overlap* values for each trial were distributed non-normally as shown in **Supplemental Figure 2**.

| 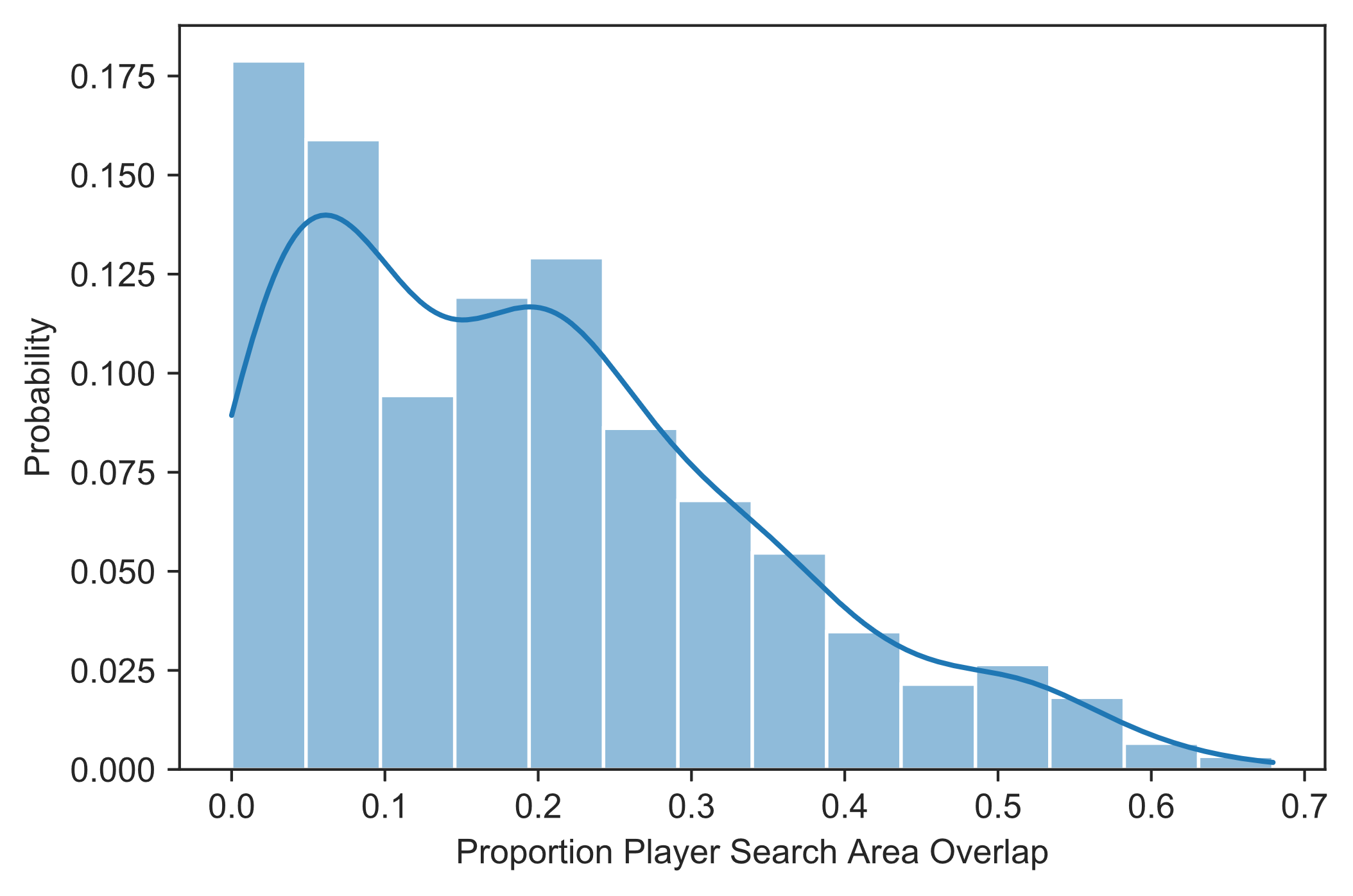  **Supplemental Figure 2. Histogram of proportion player search area overlap for all trials.** Also plotted is the kernel density estimate of the data distribution (*M* = .19, *SD* = .15). |
| --- |

To assess the effect of the experimental manipulations on the proportion of search area overlap between team members, *search area overlap* values were first transformed to an open (0,1), as opposed to closed [0,1], interval (Smithson & Verkuilen, 2006). This transformation enabled a θ-model Box-Cox regression (Box & Cox, 1964) predicting *search area overlap* from *Target Number*, *Visibility*, *HUD*, and *Session*. This regression suggested a θ of 0.46 (*SE* = 0.03, *Z* = 14.88, *p* < .001) which was used as the λ in the Box-Cox transformation of *search area overlap*. Specifically,

$$search area overlap'= \frac{{search area overlap}^{\lambda}-1}{\lambda}$$

The resulting transformed *search area overlap* was still non-normally distributed but meant that the residuals in the multilevel models (with at least main effect fixed effects) predicting transformed *search area overlap* were normally distributed, thereby facilitating the parametric analysis of the fixed effects.

The base random-intercepts beta-regression model (that is, the model with the maximal-to-converge random-effects structure but no fixed effects) fit the data acceptably (*LL* = -199.15, *AIC* = 430.30) and better than the null single-level model (LR *χ^2^*(14) = 186.80, *p* < .001, Δ*AIC* = -158.80). The model that also included the fixed effects *Target Number*, *Visibility*, *HUD*, and *Session*, as well as the random effect *team number* fit the data more appropriately than the base model (LR *χ^2^*(6) = 69.62, *p* < .001, Δ*AIC* = -57.62), and the model that included the two-way-interaction fixed effects as well fit the data more appropriately than the one-way model (LR *χ^2^*(12) = 52.39, *p* < .001, Δ*AIC* = -28.39). However, the model that also included the three-way-interaction fixed effects did not fit the data more appropriately than the two-way model (LR *χ^2^*(10) = 13.42, *p* = .201, Δ*AIC* = 6.58) and hence was rejected. No contrasts of the three-way marginal linear predictors were significant in the three-way model. Finally, five more random-slope parameters were then removed from the model (as they were too small for Stata to calculate their standard errors) and the model was refit using REML (to allow the calculation of Kenward-Roger estimates of degrees of freedom for the fixed effects).

In addition to the results in the main text, team members, on average, overlapped in their search areas more when 18 TAs were present (*M* = 0.21, *SE* = 0.01), as compared to 9 TAs (*M* = 0.17, *SE* = 0.01; *g* = 0.12, SE = 0.03, *t*(133.4) = 4.41, *p* < .001, where *g* is the estimate of the contrast {-1, 1} of the marginal linear predictions from the multilevel model predicting the Box-Cox-transformed *search overlap* scores). However, with the significant *Target Number × Visibility* interaction (*g* = -0.12, *SE* = 0.05; *t*(507.9) = -2.67, *p* = .008), this difference was only significant when participants had clear visibility of the task environment (*g* = 0.18, SE = 0.04, *t*(296.0) = 5.15, *p* < .001). When fog was present, the number of TAs that needed to be corralled and contained had no impact on the amount of overlap in participants’ search areas (*g* = 0.05, SE = 0.04, *t*(283.9) = 1.42, *p* = .312). There was no significant *Target Number × HUD* (*t*(374.5) = 0.24, *p* = .809) nor *Target Number × Session* (*F*(3, 91.8) = 1.43, *p* = .240) interactions.

To access the impact of overlap in player search areas between team members on team performance, a random-intercepts log-logistic AFT model predicting trial duration with fixed effect *search area overlap* and random effect *team number* was fit. This model fit the data more appropriately than the base log-logistic model (LR *χ^2^*(1) = 292.92, *p* < .001, Δ*AIC* = -290.92) and indicated that teams with greater *search area overlap* took significantly longer to complete trials (*b* = 2.14, *Z* = 16.67, *p* < .001). *Search area overlap* values corresponding with 2.3, 15.9, 50, 84.1, and 97.7 percentiles (which correspond to -2, -1, 0, 1, and 2 SD above the mean for a normal distribution), respectively, as well as the marginal predicted *trial duration* means and the estimated survival curves for those *search area overlap* values, are presented in **Supplemental Figure 3**.

| 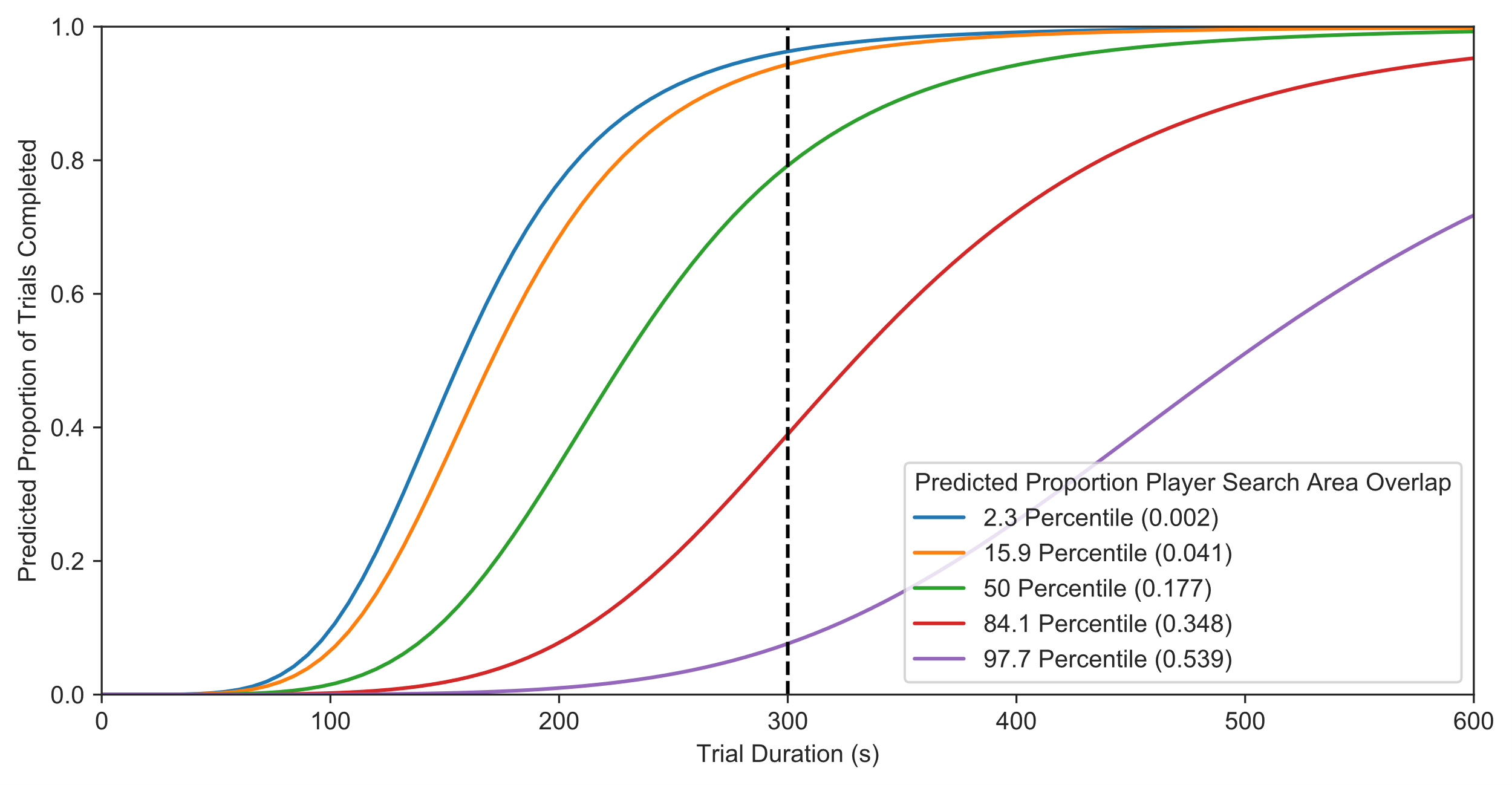  **Supplemental Figure 3. Estimated Accelerated Failure Time (AFT) Survival Curves including Proportion Search Area Overlap as a Predictor.** The y-axis represents the proportion of teams who were expected to have completed the trial by the time interval (x-axis). Separate survival curves are plotted representing the estimated proportion of search area overlap that represent the 2.3, 15.9, 50, 84.1, and 97.7 percentiles – with their associated values in parentheses. These percentiles correspond to -2, -1, 0, 1 and 2 SD above the mean for a normal distribution. As can be from the figure, teams who were expected to overlap more in where they search were also predicted to need more time to complete the trial. The vertical dotted black line represents the total time teams had to complete any given trial (300 s). |
| --- |

## Search Behavior Structure

To assess the effect of the experimental conditions on DFA_α_, several multilevel linear regression models were conducted predicting either *displacement-angle* or *head-orientation* values, with individual trials nested under players, and players nested under teams. Both measures were approximately normally distributed, as shown in **Supplemental Figure 4**.

| 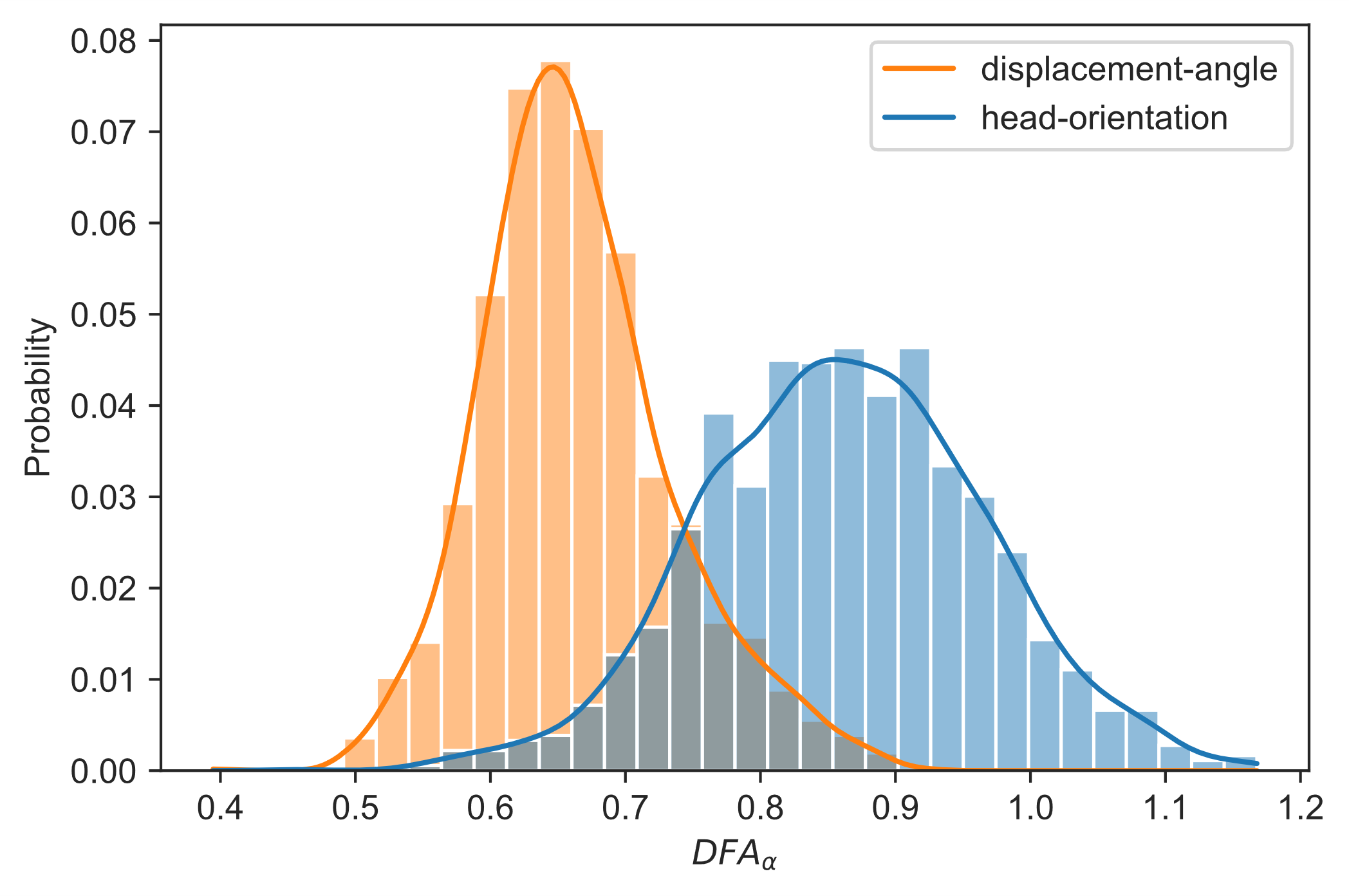  **Supplemental Figure 4. Histogram of DFA_α_ values for displacement-angle and head-orientation for all trials.** Also plotted is the kernel density estimate of the data distribution for each respective measure (displacement-angle *M* = 0.66, *SD* = 0.07; head-orientation *M* = 0.86, *SD* = 0.10). |
| --- |

### Analysis of displacement-angle DFA_α_

The base random-intercepts displacement-angle DFA_α_ model (that is, the model with the maximal-to-converge random-effects structure but no fixed effects) fit the data acceptably (*LL* = 2869.26, *AIC* = -5700.51) and better than the null single-level model ($\bar{\chi}^{2}$(17) = 1250.96, *p* < .001, Δ*AIC* = -1216.96). The model that also included the fixed effects *Target Number*, *Visibility*, *HUD*, and *Session*, and random intercepts and slopes for each *team number* fit the data more appropriately than the base model (LR *χ^2^*(6) = 37.30, *p* < .001, Δ*AIC* = -25.30). The model that included the two-way-interaction fixed effects as well fit the data more appropriately than the one-way model (LR *χ^2^*(12) = 27.77, *p* < .001, Δ*AIC* = -3.77). However, the model that also included the three-way-interaction fixed effects did not fit the data more appropriately than the two-way model (LR *χ^2^*(10) = 7.15, *p* = .711, Δ*AIC* = 12.85) and hence was rejected. None of the contrasts for the three-way marginal linear predictors in the three-way model were significant. Finally, one more random-slope parameter was then removed from the two-way model (as they were too small for Stata to calculate their standard errors) and the model was refit using REML (to allow the calculation of Kenward-Roger estimates of degrees of freedom for the fixed effects).

In addition to the results in the main text, team members had lower displacement-angle DFA_α_ values when corralling 18 (*M* = 0.654, *SE* = 0.002), compared to 9 TAs (*M* = 0.674, *SE* = 0.002; *g* = -0.020, *SE* = 0.003, *t*(10.2) = -6.78, *p* < .001, where *g* is the estimate of the contrast {-1, 1} of the marginal linear predictions in the displacement-angle DFA_α_ multilevel model). There was no significant *Target Number × HUD* (*g* = -0.004, *SE* = 0.004, *t*(169.6) = -0.85, *p* =.385), *Target Number × Visibility* (*t*(177.4) = 1.91, *p* = .058, nor *Target Number × Session* (*F*(3, 442.8) = 2.50, *p* =.059) interactions.

### Analysis of head-orientation DFA_α_

The base random-intercepts displacement-angle DFA_α_ model (that is, the model with the maximal-to-converge random-effects structure but no fixed effects) fit the data acceptably (*LL* = 2308.47, *AIC* = -4540.95) and better than the null single-level model ($\bar{\chi}^{2}$(36) = 1516.77, *p* < .001). The model that also included the fixed effects *Target Number*, *Visibility*, *HUD*, and *Session*, and random intercepts and slopes for each *team number* fit the data more appropriately than the base model (LR *χ^2^*(6) = 71.74, *p* < .001, Δ*AIC* = -59.74). The model that included the two-way-interaction fixed effects as well fit the data more appropriately than the one-way model (LR *χ^2^*(12) = 60.41, *p* < .001, Δ*AIC* = -96.15), but the model that also included the three-way-interaction fixed effects did not fit the data more appropriately than the two-way model (LR *χ^2^*(10) = 16.93, *p* = .076, Δ*AIC* = 3.06). However, the contrast of the *HUD × Visibility × Session* marginal linear predictors in the three-way model was significant (*χ^2^*(3) = 9.80, *p* = .020). When these parameters were added to the two-way model, they significantly improved the model fit (LR *χ^2^*(3) = 9.80, *p* = .020, Δ*AIC* = -3.80) and were accepted in the model. Ten more random-slope parameters were then removed from the model (as they were too small for Stata to calculate their standard errors) and the model was refit using REML (to allow the calculation of Kenward-Roger estimates of degrees of freedom for the fixed effects). Finally, three more random-slopes parameters were then removed under the same decision criterion.

In addition to the results in the main text, players had lower head-orientation DFA_α_ values when the task required the containment of 18 (*M* = 0.849, *SE* = 0.003), compared to 9 TAs (*M* = 0.875, *SE* = 0.004; *g* = -0.025, *SE* = 0.004, *t*(9.2) = -6.65, *p* < .001, where *g* is the estimate of the contrast {-1, 1} of the marginal linear predictions in the head-orientation DFA_α_ multilevel model). The significant *Target Number × Visibility* interaction (*t*(21.2) = 3.07, *p* = .006) indicated that although head-orientation DFA_α_ was lower when participants were tasked to search and contain 18, compared to 9, TAs regardless of environment visibility (fog: *g* = -0.016, *SE* = 0.005, *t*(9.8) = -3.55, *p* = .011; clear visibility: *g* = -0.034, *SE* = 0.005, *t*(20.0) = -6.98, *p* < .001), this effect was greater when the environment had no fog (*g* = -0.018, *SE* = 0.006, *t*(21.2) = -3.06, *p* = .006). Examining the interaction from the other perspective, teams exhibited significantly lower head-orientation DFA_α_ values when there was fog only when there were 9 TAs (*g* = -0.035, *SE* = 0.012, *t*(14.4) = -2.93, *p* = .021). When there were 18 TAs, there was no significant difference (*g* = -0.018, *SE* = 0.012, *t*(14.6) = -1.45, *p* = .334).

There was also a significant *Target Number × Session* interaction (*F*(3, 93.2) = 5.30, *p* = .002), such that head-orientation DFA_α_ values were lower when participants were tasked to contain 18 TAs, but only during the second (*g* = -0.024, *SE* = 0.006, *t*(20.35) = -3.73, *p* = .005), third (*g* = -0.030, *SE* = 0.006, *t*(27.2) = -4.34, *p* < .001), and fourth and final (*g* = -0.039, *SE* = 0.006, *t*(55.2) = -6.59, *p* < .001) sessions (first session: *g* = -0.008, *SE* = 0.006, *t*(64.5) = -1.33, *p* = .756). Examining this interaction from the other direction, head-orientation DFA_α_ values were greater in the final session than in the first session regardless of the number of TAs that needed to be contained (9 TAs: *g* = 0.071, *SE* = 0.012, *t*(11.3) = 5.82, *p* < .001; 18 TAs: *g* = 0.041, *SE* = 0.012, *t*(11.3) = 3.32, *p* = .013). However, this improvement was greater when there were 9 compared to 18 TAs (*g* = 0.031, *SE* = 0.008, *t*(1467.91) = 3.93, *p* < .001). The *Target Number × HUD* interaction was not significant (*t*(256.9) = -0.11, *p* = .909).

## Verbal Communication

Team verbal communication was also investigated, which was quantified as the average number of words spoken by the team (collectively) per second (*word count rate*). Due to technical difficulties in audio recording, *word count rate* could not be calculated for 5 (0.83%) of the 604 trials submitted for analyses. The *word count rate* values for each trial were positively skewed as shown in **Supplemental Figure 5**. However, the residuals in the subsequently described multilevel models were approximately normally distributed, as determined with visual inspection of the histogram and Q-Q plot of those residuals.

| 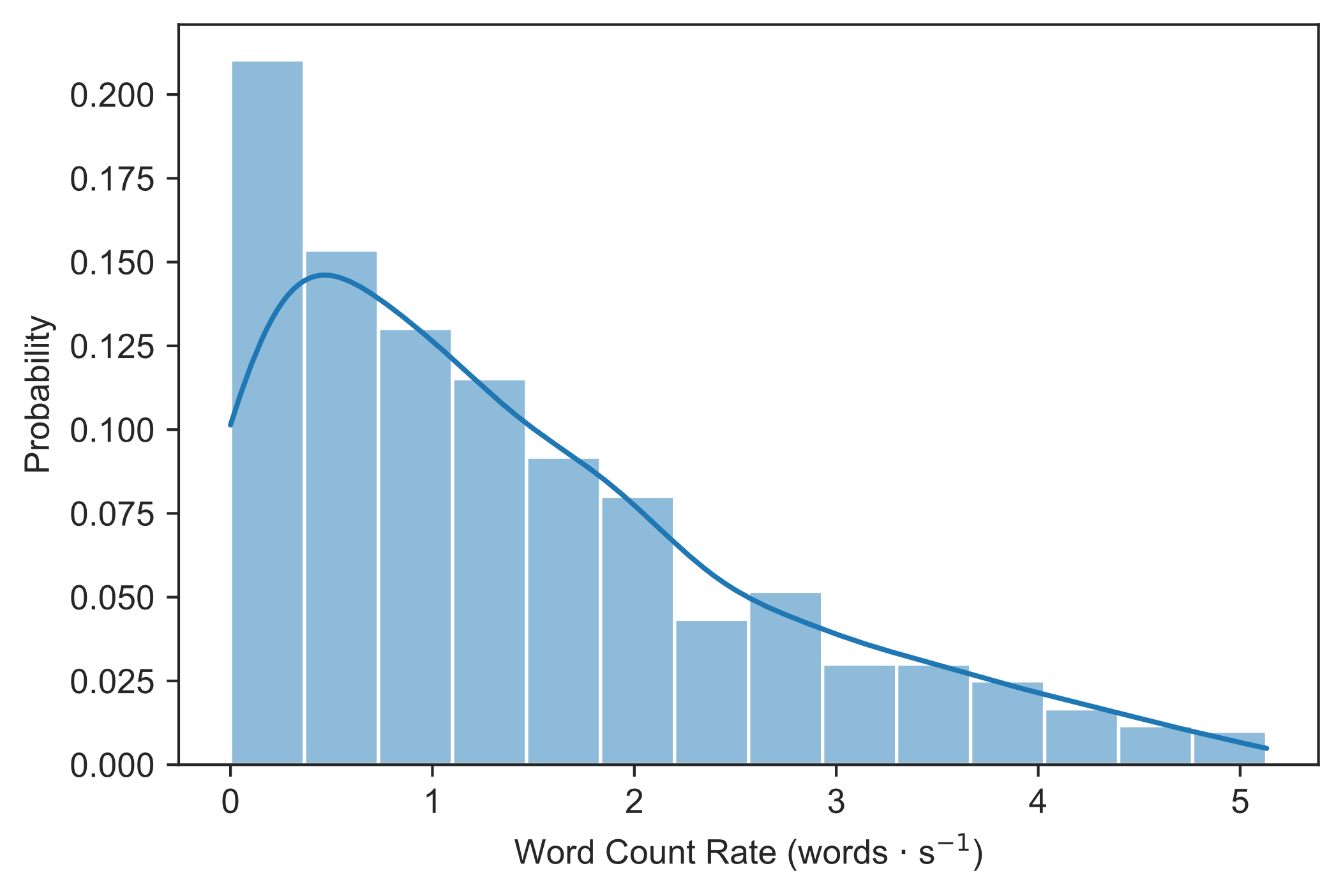  **Supplemental Figure 5. Histogram of team word count rate for all trials.** Also plotted is the kernel density estimate of the data distribution (*M* = 1.41, *SD* = 1.19). |
| --- |

The base random-intercepts *word count rate* model (that is, the model with the maximal-to-converge random-effects structure but no fixed effects) fit the data acceptably (*LL* = -477.52, *AIC* = 993.04) and better than the null single-level model ($\bar{\chi}^{2}$(17) = 948.55, *p* < .001, Δ*AIC* = -914.55). The model that also included the fixed effects *Target Number*, *Visibility*, *HUD*, and *Session*, and random intercepts and slopes for each *team number* fit the data more appropriately than the base model (LR *χ^2^*(6) = 15.06, *p* = .020, Δ*AIC* = -3.06). The model that included the two-way-interaction fixed effects as well fit the data more appropriately than the one-way model (LR *χ^2^*(9) = 26.27, *p* = .002, Δ*AIC* = -8.27). However, the model that also included the three-way-interaction fixed effects did not fit the data more appropriately than the two-way model (LR *χ^2^*(11) = 12.55, *p* = .324, Δ*AIC* = 9.45) and hence was rejected. None of the contrasts for the three-way marginal linear predictors in the three-way model were significant. Finally, six more random-slope parameters were then removed from the two-way model (as they were too small for Stata to calculate their standard errors) and the model was refit using REML (to allow the calculation of Kenward-Roger estimates of degrees of freedom for the fixed effects).

On average, teams uttered fewer words per second when the task required the containment of 18 (*M* = 1.36, *SE* = 0.07), compared to 9 TAs (*M* = 1.47, *SE* = 0.07; *g* = -0.12, *SE* = 0.05, *t*(9.3) = -2.56, *p* = .030, where *g* is the estimate of the contrast {-1, 1} of the marginal linear predictions in the word count rate multilevel model). Similarly, teams uttered fewer words per second when the HUD was available (*M* = 1.26, *SE* = 0.07) compared to when they only had access to a compass (*M* = 1.56, *SE* = 0.07; *g* = -0.31, *SE* = 0.09, *t*(9.5) = -3.56, *p* = .006). However, a significant *Target Number* *× HUD* interaction (*g* = -0.20, *SE* = 0.08, *t*(21.1) = -2.51, *p* = .020) indicated that when teams had to contain 18 TAs, the presence of the HUD (*M* = 1.14, *SE* = 0.09) significantly decreased the amount of team verbal communication compared to when teams had access to a compass (*M* = 1.57, *SE* = 0.10; *g* = -0.41, *SE* = 0.10, *t*(12.9) = -4.14, *p* = .002). There was no impact on whether the HUD was available when only 9 TAs were present (*M_compass_* = 1.55, *SE_compass_* = 0.09, *M_HUD_* = 1.39, *SE_HUD_* = 0.10; *g* = -0.21, *SE* = 0.09, *t*(12.2) = -2.24, *p* = .089). Considering the interaction from the other direction, when teams only had access to a compass, there was no difference in *word count rate* as a function of the number of TAs that needed to be contained (*g* = -0.02, *SE* = 0.06, *t*(18.7) = -0.30, *p* > .999). However, when teams did have access to a HUD, teams communicated less when 18, as opposed to 9 TAs, had to be contained (*g* = -0.22, *SE* = 0.07, *t*(10.2) = -3.30, *p* = .016).

Additionally, there was a significant *Visibility × HUD* interaction (*g* = -0.29, *SE* = 0.08, *t*(313.3) = -3.81, *p* < .001). When teams were exposed to environmental fog, the presence of the HUD decreased the amount of verbal communication (*M* = 1.26, *SE* = 0.10) compared to when teams only had access to the compass (*M* = 1.68, *SE* = 0.09; *g* = -0.45, *SE* = 0.10, *t*(14.1) = -4.73, *p* < .001). When teams had a clear view of the environment, there was no difference in verbal communication between when teams had a compass (*M* = 1.44, *SE* = 0.09) or HUD (*M* = 1.27, *SE* = 0.10; *g* = -0.16, *SE* = 0.09, *t*(12.8) = -1.74, *p* = .21). Exploring the interaction from the other direction, environmental visibility had no impact on verbal communication when teams had access to the HUD (*g* = 0.04, *SE* = 0.09, *t*(15.3) = 0.42, *p* > .99). However, when teams only had access to the compass, more verbal communication was observed when teams were exposed to fog (*g* = 0.26, *SE* = 0.08, *t*(13.5) = 3.12, *p* = .016).

There were no significant main effects of *Visibility* (*g* = 0.11, *SE* = 0.07, *t*(9.1) = 1.48, *p* = .173) nor *Session* (*F*(3, 13.6) = 1.83, *p* = .189). The two-way *Target Number* × *Visibility* (*g* = 0.002, SE = 0.07, *t*(492.6) = 0.02, *p* = .983), *Target Number* × *Session* (*F*(3, 495.0) = 1.59, *p* = .190), *HUD* × *Session* (*F*(3, 47.9) = 1.09, *p* = .361), and *Visibility* × *Session* (*F*(3, 44.6) = 1.25, *p* = .303) interactions were all non-significant.

# References

Barr, D. J., Levy, R., Scheepers, C., & Tily, H. J. (2013). Random effects structure for confirmatory hypothesis testing: Keep it maximal. *Journal of Memory and Language*, *68*(3), 255–278. https://doi.org/10.1016/j.jml.2012.11.001

Box, G. E. P., & Cox, D. R. (1964). An Analysis of Transformations. *Journal of the Royal Statistical Society. Series B (Methodological)*, *26*(2), 211–252. http://www.jstor.org/stable/2984418

Kenward, M. G., & Roger, J. H. (1997). Small Sample Inference for Fixed Effects from Restricted Maximum Likelihood. *Biometrics*, *53*(3), 983. https://doi.org/10.2307/2533558

Meteyard, L., & Davies, R. A. I. (2020). Best practice guidance for linear mixed-effects models in psychological science. *Journal of Memory and Language*, *112*, 104092. https://doi.org/10.1016/J.JML.2020.104092

Smithson, M., & Verkuilen, J. (2006). A better lemon squeezer? Maximum-likelihood regression with beta-distributed dependent variables. *Psychological Methods*, *11*(1), 54–71. https://doi.org/10.1037/1082-989X.11.1.54
