# Supplementary figures and images for: Assessing Team Effectiveness by How Players Structure Their Search in a First‐Person Multiplayer Video Game
Source: Cogn Sci. 2022 Oct 17;46(10):e13204. doi: 10.1111/cogs.13204 (PMC9787020; doi:10.1111/cogs.13204)

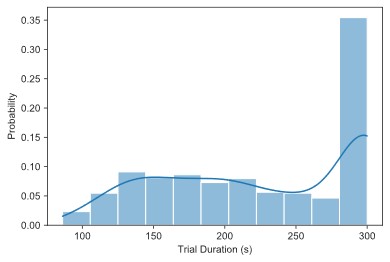

Supplement: Supplementary file 2 — Supplemental Figure 1. Histogram of trial duration for all trials. [file COGS-46-e13204-s007.jpg]

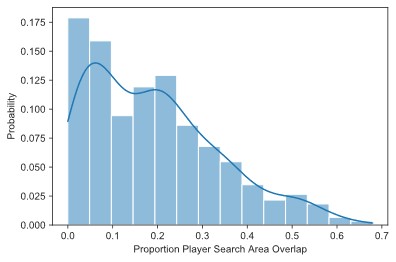

Supplement: Supplementary file 3 — Supplemental Figure 2. Histogram of proportion player search area overlap for all trials. [file COGS-46-e13204-s005.jpg]

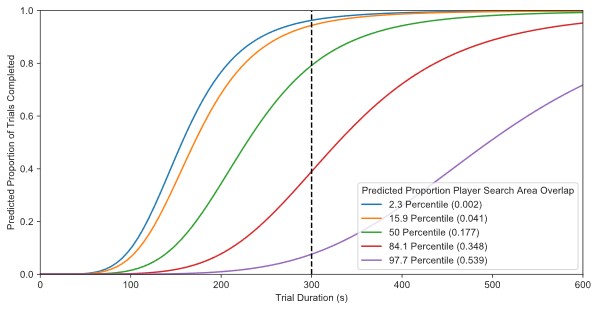

Supplement: Supplementary file 4 — Supplemental Figure 3. Estimated accelerated failure time (AFT) survival curves including proportion search area overlap as a predictor. [file COGS-46-e13204-s002.jpg]

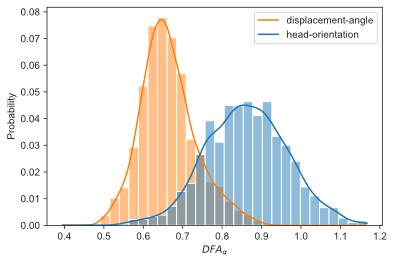

Supplement: Supplementary file 5 — Supplemental Figure 4. Histogram of DFAα values for displacement‐angle and head‐orientation for all trials. [file COGS-46-e13204-s006.jpg]

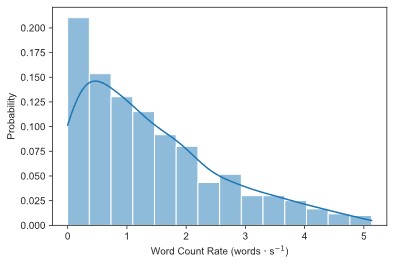

Supplement: Supplementary file 6 — Supplemental Figure 5. Histogram of team word count rate for all trials. [file COGS-46-e13204-s004.jpg]
