# Supplementary material for: Assessing Team Effectiveness by How Players Structure Their Search in a First‐Person Multiplayer Video Game
Source: Cogn Sci. 2022 Oct 17;46(10):e13204. doi: 10.1111/cogs.13204 (PMC9787020; doi:10.1111/cogs.13204)
Supplement: Supplementary file 7 — Supplemental Tables. Final Models. [file COGS-46-e13204-s001.docx]

| DV | Random-effect parameters (Team) | Random-effect parameters (Player) | Fixed-effect parameters | Model fit | | | | Residual intraclass correlation | | | | | |
| --- | --- | --- | --- | --- | --- | --- | --- | --- | --- | --- | --- | --- | --- |
|  |  |  |  | AIC | BIC | LL | df | Level | ICC | | SE | [95% CI] | |
| Head-orientation DFA_α_ | Intercepts;  All 1-way main effect slope parameters;  Target Number × Visibility, HUD × Visibility, Visibility × Session 4;  Target Number × HUD × Session 2, Target Number × Visibility × Session 2, Target Number × Visibility × Session 3, HUD × Visibility × Session 3 | Intercepts;  All 1-way main effect slope parameters;  HUD × Visibility, HUD × Session 2;  Target Number × HUD × Session 3, HUD × Visibility × Session 3. | Intercept;  All 1-way main effect parameters;  All 2-way interaction parameters;  HUD × Visibility × Session 2, HUD × Visibility × Session 3, HUD × Visibility × Session 4. | -4485.37 | -4199.26 | 2294.69 | 52 | Team | .06 | | .13 | < .01, .89 | |
|  |  |  |  |  |  |  |  | Team \| Player | .60 | | .07 | .45, .73 | |
| Displacement-angle DFA_α_ | All 1-way main effect slope parameters; | Intercepts;  All 1-way main effect slope parameters;  HUD × Session 2. | Intercept;  All 1-way main effect parameters;  All 2-way interaction parameters. | -5566.82 | -5368.74 | 2819.41 | 36 | Team | < .01 | < .01 | | | < .01, < .01 |
|  |  |  |  |  |  |  |  | Team \| Player | .59 | | .07 | .45, .72 | |

| DV | Random-effect parameters (Team) | Random-effect parameters (Player) | Fixed-effect parameters | Model fit | | | | Residual intraclass correlation | | | |
| --- | --- | --- | --- | --- | --- | --- | --- | --- | --- | --- | --- |
|  |  |  |  | AIC | BIC | LL | df | Level | ICC | SE | [95% CI] |
| Box-Cox-transformed Proportion Search Area Overlap  (i.e., $\frac{\mathrm{value}^{0.46}-1}{0.46}$ ) | Intercepts;  HUD, Visibility, Session 2, Session 4;  Target Number × Session 4;  Target Number × HUD × Session 3, HUD × Visibility × Session 4;  Target Number × HUD × Visibility × Session 2. | None (DV was at the Team level) | Intercept;  All 1-way main effect parameters;  All 2-way interaction parameters. | 418.08 | 550.19 | -179.04 | 30 | Team | .26 | .11 | .11, .51 |
| Word Count Rate | Intercepts;  All 1-way main effect slope parameters;  Target Number × HUD, HUD × Session 4, Visibility × Session 3;  HUD × Visibility × Session 4. | None (DV was at the Team level) | Intercept;  All 1-way main effect parameters;  All 2-way interaction parameters. | 1025.79 | 1162.04 | -481.89 | 31 | Team | .87 | .06 | .72, .94 |

| DV | Random-effect parameters (Team) | Random-effect parameters (Player) | Fixed-effect parameters | Model fit | | | | Residual intraclass correlation | | | |
| --- | --- | --- | --- | --- | --- | --- | --- | --- | --- | --- | --- |
|  |  |  |  | AIC | BIC | LL | df | Level | ICC | SE | [95% CI] |
| Log of Trial Duration (modelled using a log-logistic AFT survival time multilevel model, scale parameter = 0.12) | Intercepts. | None (DV was at the Team level) | Intercept;  All 1-way main effect parameters;  All 2-way interaction parameters. | 4417.37 | 4509.84 | -2187.37 | 21 | Team | .10 | .04 | .02, .19 |
| Log of Trial Duration (using same log-logistic AFT survival model, scale parameter = 0.21) | Intercepts. | None (DV was at the Team level) | Intercept;  Displacement-angle DFA_α_. | 4928.36 | 4945.98 | -2460.18 | 4 | Team | .04 | .02 | < .01, .07 |
| Log of Trial Duration (using same log-logistic AFT survival model, scale parameter = 0.16) | Intercepts. | None (DV was at the Team level) | Intercept;  Head-orientation DFA_α_. | 4639.54 | 4657.15 | -2315.77 | 4 | Team | .12 | .05 | .02, .21 |
| Log of Trial Duration (using same log-logistic AFT survival model, scale parameter = 0.19) | Intercepts. | None (DV was at the Team level) | Intercept;  Proportion Search Area Overlap. | 4783.82 | 4801.44 | -2387.91 | 4 | Team | .02 | .01 | < .01, .04 |
